# Exosomes containing miR-152-3p targeting FGFR3 mediate SLC7A7-induced angiogenesis in bladder cancer

**DOI:** 10.1038/s41698-025-00859-z

**Published:** 2025-03-12

**Authors:** Chun Cao, Yu Wang, Xiaolin Deng, Xinlei Zhao, Yuwen Chen, Wanlong Tan, Fan Deng, Fei Li

**Affiliations:** 1 Department of Urology, Nanfang Hospital, Southern Medical University, Guangzhou, Guangdong People’s Republic of China; 2 Department of Urology, Ganzhou People’s Hospital, Ganzhou, People’s Republic of China; 3 Department of Cell Biology, School of Basic Medical Sciences, Southern Medical University, Guangzhou, People’s Republic of China

**Keywords:** Tumour angiogenesis, Bladder cancer

## Abstract

Bladder cancer (BCa) is a prevalent malignancy with a poor prognosis. SLC7A7 has been linked to BCa progression and angiogenesis, but its specific role remains unclear. We established a SLC7A7-knockdown BCa cell line to investigate its effects on angiogenesis. In vivo experiments assessed tumor vascularization, while in vitro studies explored exosome involvement. MiRNA sequencing identified miR-152-3p as a key regulator. Further investigation using dual-luciferase reporter assays, qRT-PCR, and Western blot revealed that miR-152-3p inhibits the expression of FGFR3 by binding to its 3’ UTR. Meanwhile, functional assays, including angiogenesis assays, Transwell assays, and wound healing assays, were performed to evaluate the effects of miR-152-3p on angiogenesis. We confirmed the significant role of SLC7A7 in BCa progression, specifically in promoting angiogenesis, through the involvement of exosomes and the regulatory axis of miR-152-3p/ FGFR3. Targeting FGFR3 might be a promising strategy to reverse control BCa progression for an improved prognosis.

## Introduction

Bladder cancer (BCa) stands as one of the most prevalent malignancies affecting the urinary system^1^. Notably, in 15–20% of patients, the development of muscle-invasive BCa (MIBC) is observed, contributing to increased mortality rates^2–4^. Despite commendable advances in medical methodologies and therapeutic paradigms, the overall survival (OS) for BCa remains unchanged. Within this intricate landscape, targeted therapy emerges as a novel and promising approach for addressing BCa^5,6^. However, the specific sites and mechanisms suitable for targeted therapy need to be further explored. Consequently, a comprehensive understanding of the molecular mechanisms governing BCa becomes paramount for advancing therapeutic strategies and ultimately enhancing patient prognosis.

Tumor angiogenesis plays a crucial role in the progression of BCa and serves as a rational target for anti-cancer therapies^7^. Additionally, the activity of tumor angiogenesis is a vital parameter for assessing the behavior and therapeutic outcomes of BCa. However, despite these insights, the combination of vascular endothelial growth factor/ VEGF receptor (VEGF/VEGFR) inhibitors has not shown an improvement in OS when compared to chemotherapy alone^8^. This suggests that the effectiveness and indications for anti-angiogenic therapies in clinical practice remain limited. Consequently, there is a continuing need to identify the unique characteristics of tumor endothelium that are essential for pathological angiogenesis.

SLC7A7, a crucial gene in the solute carrier family 7 (SLC7), is essential for the transport of cationic amino acids like arginine and functions alongside SLC3A2/4F2hc to facilitate this process^9^. Recent studies suggest that SLC7A7 expression is linked to immune infiltration and poor prognosis in NSCLC^10^. Elevated SLC7A7 levels in tumor cells can lead to decreased intracellular arginine, promoting cell migration and invasion while inhibiting apoptosis^11^. Conversely, this overexpression also increases arginine levels in the microenvironment, leading to immune cell infiltration^10^. Additionally, SLC7A7 may indirectly contribute to endothelial cell dysfunction^12^. LAT1, a protein encoded by SLC7A5 and a member of the same family as SLC7A7, is critical for tumor angiogenesis by regulating cell proliferation, translation, and pro-angiogenic VEGF-A signaling^13^. However, the exact relationship between SLC7A7 and tumor angiogenesis remains unclear.

Angiogenesis is an intricate process governed by numerous factors, among which the tumor microenvironment (TME) plays a pivotal regulatory role^14,15^. This TME constitutes a complex network of soluble substances, including tumor cells, surrounding cells, and secreted cytokines^16^. Notably, the emerging significance of exosomes in the TME has garnered considerable attention in recent years^17,18^. Exosomes, a class of extracellular vesicles containing biologically active molecules such as mRNA, proteins, and miRNA, serve as crucial mediators in cell-to-cell communication^19,20^. In the context of the TME, the release of exosomes has been confirmed to be closely related to angiogenesis, offering an additional layer of regulation^21^. For instance, the release of exosome-derived glutamine--fructose-6-phosphate transaminase 1 (GFAT1) from BCa cells has been shown to induce metabolic reprogramming and alter glycosylation levels in endothelial cells, thereby promoting neovascularization in BCa^22^. However, the role and potential mechanism of exosomes in modulating angiogenesis within the TME remain poorly understood.

In this study, we observed that members of the SLC7 are significantly up-regulated in MIBC, and this overexpression was strongly associated with poor prognosis and tumor progression. Employing gene set enrichment analysis (GSEA), we predicted the biological function of SLC7A7, revealing a strong association with angiogenesis. Both in vivo and in vitro experiments demonstrated that SLC7A7 actively promotes BCa angiogenesis through exosomes. Through further exploration of miRNA sequencing, we found that miR-152-3p is a key factor related to angiogenesis. Notably, we found that miR-152-3p targets fibroblast growth factor 3 (FGFR3) in human endothelial cells, establishing FGFR3 as a functional downstream target of miR-152-3p. In vitro validations confirmed the ability of the exosome-mediated miR-152-3p/FGFR3 axis to inhibit angiogenesis, as well as endothelial cell invasion and metastasis. Our results underscore the role of BCa cells with elevated SLC7A7 expression in promoting angiogenesis by down-regulating exosome miR-152-3p levels. Furthermore, these findings suggest that FGFR3 is a potential target for anti-angiogenic therapy in BCa. Collectively, this study provides novel insights that could inform the development of innovative treatment strategies for BCa.

## Results

### SLC7A7 is highly expressed in BCA tissues and associated with poor prognosis

**Fig. 1:**
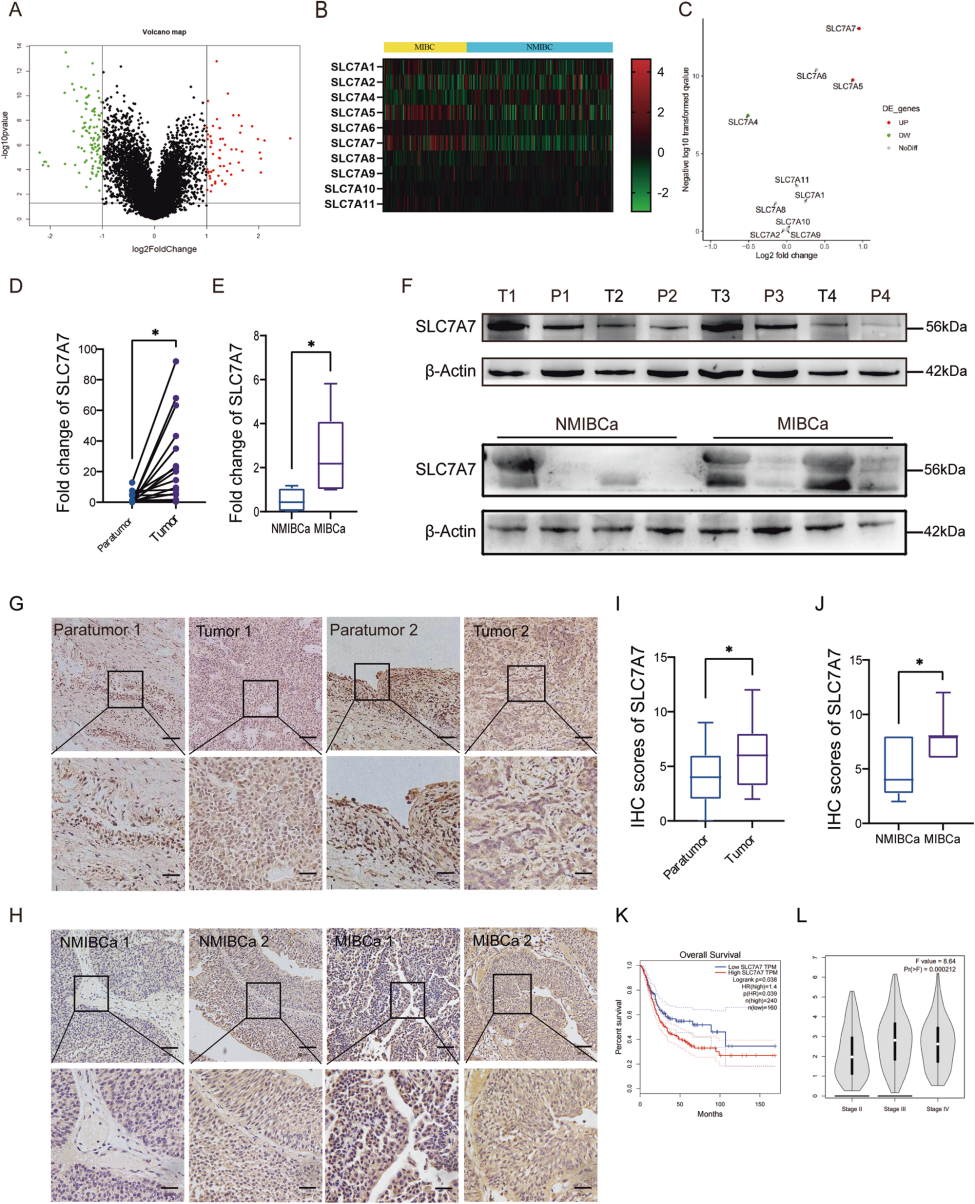
SLC7A7 in MIBCa high expression and is associated with poor prognosis. **A** Volcano plot of NMIBCa and MIBCa differential genes in the GEO dataset. **B** Heat map of SLC7A family expression in NMIBCa and MIBCa. **C** Volcano plot of SLC7A family expression in NMIBCa and MIBCa. **D**, **E** mRNA expression of SLC7A7 in BCa tissues and paratumor tissues to cancer, in NMIBCa tissues and MIBCa tissues. **F** Protein expression of SLC7A7 in BCa tissues and paratumor tissues adjacent to cancer, in NMIBCa tissues and MIBCa tissues. **G**, **I** Immunohistochemical results of SLC7A7 in BCa tissues and paratumor tissues to cancer and their scoring results. Scale bar: 50 μm and 20 μm. **H**, **J** Immunohistochemical results of SLC7A7 in NMIBCa tissues and MIBCa tissues and their scoring results. **K** The Kaplan–Meier survival analysis of SLC7A7. **L** Correlation between SLC7A7 expression and BCa stage in the TCGA dataset. Statistical analyses were performed using a two-tailed Student’s *t*-test, with significance levels indicated as *P < 0.05, representing significant differences between groups.

The gene set GSE32548, housed in the Geo Public database, underwent meticulous analysis and was partitioned into two distinct groups: muscle-invasive BCa (MIBCa) and non-MIBCa (NMIBCa) Group, depending on the occurrence of muscle aggression. Digging deeper into the differences between the two groups, we found that the MIBCa group had 58 up-regulated genes and 102 down-regulated genes, in stark contrast to the NMIBCa group (Fig. 1A). To gain further insight, we conducted an Encyclopedia of Genes and Genomes (KEGG) enrichment analysis on the up-regulated genes, which revealed a notable enrichment in protein digestion and absorption (Fig. S1). This finding implies that amino acid metabolism may play a crucial role in the progression of MIBCa. Bolstered by these findings, our attention was drawn to the captivating world of the amino acid transporter protein family-SLC7A family^23^. Presently, it has been reported that this protein family is closely related to the occurrence and development of tumors, showing different expression levels and modes of action in different types of tumors^24^. Our investigation encompassed an in-depth analysis of the transcription levels of the ten principal members of this protein family, drawing from the TCGA database (Fig. 1B). Intriguingly, the results unfurled the exceedingly high expression of SLC7A7 in MIBCa (Fig. 1C, D). The expression of SLC7A7 was increased in higher-grade BCa tissues, especially in TIII stage and TVI stage (Fig. 1L). In addition, both RNA and protein levels of SLC7A7 were significantly elevated in BCa samples, and further elevated in MIBCa tumor tissues (Fig. 1E–J). The Kaplan–Meier survival analysis also demonstrated that BCa patients with high SLC7A7 expression had significantly lower OS (Fig. 1K), indicating an association between high SLC7A7 levels and poor prognosis in BCa. Driven by this revelation, we selected SLC7A7 as the prime focal point for our subsequent research endeavors.

### SLC7A7 is a potential promoter of angiogenesis

**Fig. 2:**
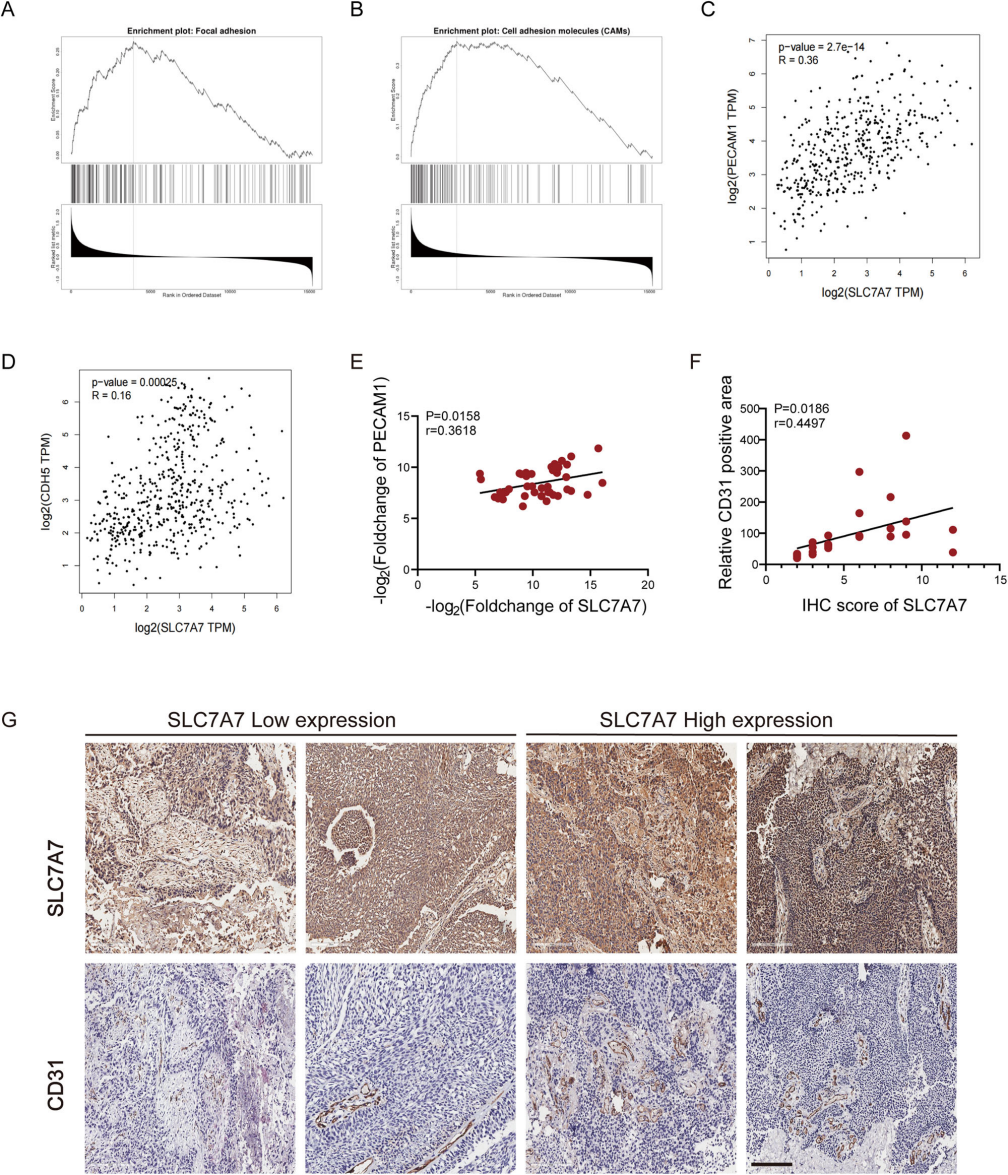
SLC7A7 was positively correlated with angiogenesis. **A**, **B** Functional enrichment of SLC7A7 expression-related genes in cell surface adhesion and cell adhesion molecules; (**C**) Pearson correlation analysis between PECAM1 and SLC7A7 levels. **D** Pearson correlation analysis between CDH5 and SLC7A7 levels. **E** Pearson correlation analysis between SLC7A7 and CD31 mRNA levels. **F**, **G** SLC7A7 and CD31 expression in BCa tissues was assessed by IHC (**G**), and the Pearson correlation analysis between CD31 positive area with SLC7A7 IHC (**F**). Scale bar: 200 μm.

To elucidate the biological role of SLC7A7 in BCa, we conducted a comprehensive analysis of SLC7A7 expression using GSEA. As shown in Fig. 2A, B, we found that genes positively associated with SLC7A7 are enriched in processes associated with cell surface adhesion and cell adhesion molecules. In addition, we analyze the correlation between endothelial cell markers, such as platelet endothelial cell adhesion molecule-1 (PECAM1/CD31) and vascular endothelial cadherin (Ve-cadherin, CDH5), and the expression of SLC7A7. Our findings indicate that either PECAM1 or CDH5 shows a positive correlation with SLC7A7 (Fig. 2C, D). This evidence strongly suggests that SLC7A7 may play a crucial role in regulating BCa progression by actively promoting angiogenesis.

Furthermore, we collected clinical tissue samples from 44 BCa patients to assess mRNA levels and performed immunohistochemistry (IHC) analyses. Pearson correlation analysis indicated that SLC7A7 and CD31 expression were positively correlated in BCa tissues (Fig. 2E). The SLC7A7 pathological score served as the benchmark for evaluating the expression level of SLC7A7. Moreover, the positive area of CD31 showed a statistically significant positive correlation with the IHC score of SLC7A7 (Fig. 2F, G). These findings clearly establish a positive association between SLC7A7 expression and vascular density, suggesting that SLC7A7 may play a crucial role in angiogenesis.

### Silencing SLC7A7 inhibits angiogenesis in vivo

To investigate the biological significance of SLC7A7, we ascertained the expression profile of SLC7A7 within BCa cell lines, namely T24, UMUC-3, and SW780. Notably, T24 and SW780 cells exhibited an elevated expression of SLC7A7 at both protein and mRNA levels (Fig. S2A, B).

Subsequently, we engineered stable cell lines, designated as SW780-shSLC7A7#1, SW780-shSLC7A7#2, T24-shSLC7A7#1, and T24-shSLC7A7#2, in which SLC7A7 was effectively silenced through lentiviral transfection of SW780 and T24 cells. This silencing was carefully confirmed by qRT-PCR and Western blotting, as shown in Fig. S2C-F, which demonstrated a down-regulation of both mRNA and protein levels of SLC7A7 in these cell lines.

**Fig. 3:**
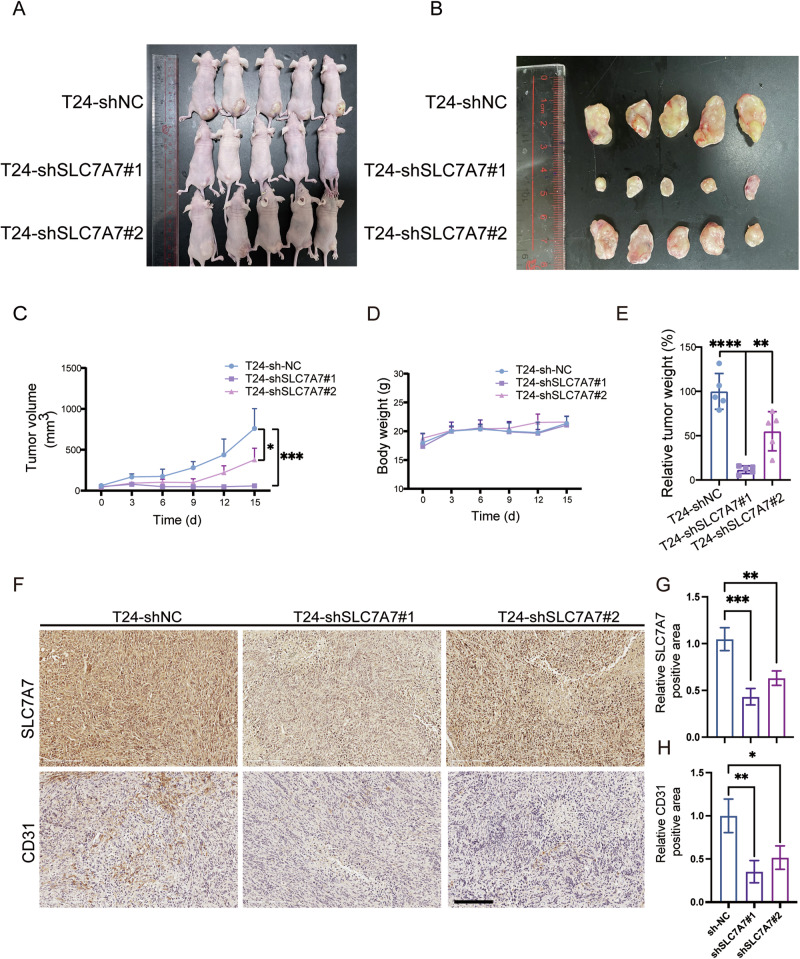
SLC7A7 promotes ss angiogenesis in vivo. **A** Construction of subcutaneous transplantation tumor model in nude mice (*n* = 5 per group). **B** Neoplasia of nude mice 15 days following injection of T24-shNC, T24-shSLC7A7#1, and T24-shSLC7A7#2 (*n* = 5 per group). **C**, **D** Tumor volumes and body weight were measured at 6 time points. **E** Tumor weights were measured at the endpoint. **F**–**H** The expression of SLC7A7 and CD31 in tumor tissues of nude mice was checked by IHC and was quantified by ImageJ (**G**, **H**). Scale bar: 200 μm. Statistical analyses were performed using a two-tailed Student’s *t*-test, with significance levels indicated as *P < 0.05, **P < 0.01, ***P < 0.001, and ****P < 0.001, representing significant differences between groups.

We explored the impact of SLC7A7 on angiogenesis in vivo through a nude mouse model of subcutaneous tumorigenesis. Following the silencing of SLC7A7, the tumors in the knockdown groups exhibited markedly diminished volume, weight, and size in comparison to the control group (Fig. 3A–E). Concurrently, there was no noteworthy difference in body weight observed across the groups during the feeding period (Fig. 3D). Immunohistochemical staining revealed a significant decrease in CD31 expression in the SLC7A7 knockdown group, suggesting a reduction in tumor angiogenesis following SLC7A7 knockdown (Fig. 3F–H). Collectively, these findings strongly indicate that the silencing of SLC7A7 exerts inhibitory effects on angiogenesis, thereby impeding tumor progression in vivo.

### SLC7A7-mediated exosomes are involved in BCA angiogenesis

**Fig. 4:**
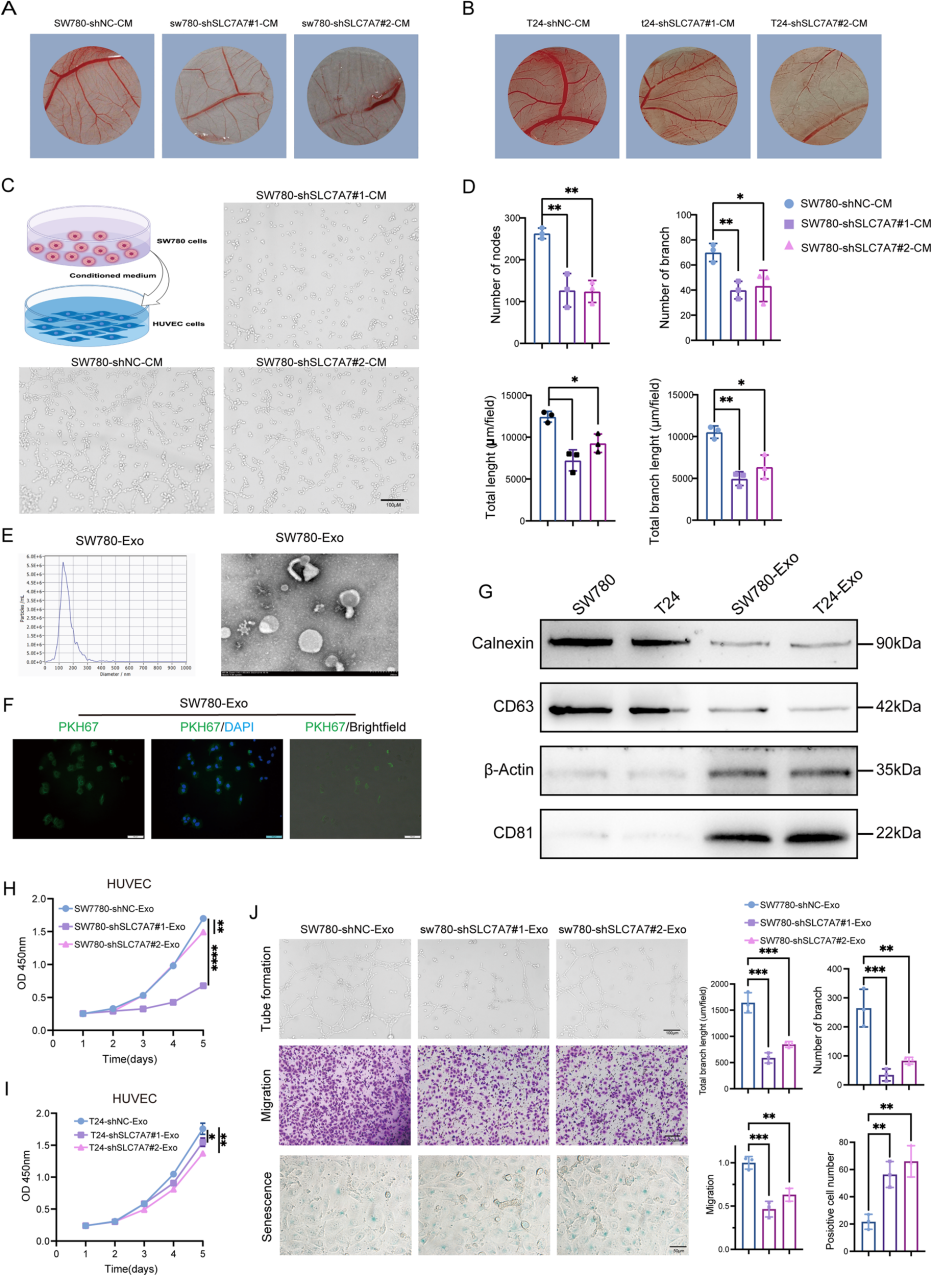
SLC7A7 promotes BCa angiogenesis through exosomes. **A**, **B** Angiogenesis was measured by CAM assays after treatment with SW780 and T24 CM. **C**, **D** Angiogenesis was assessed by tube formation assay with quantitative analysis (right panel). **E** The size of the SW780 cells-derived exosomes was analyzed by NTA, and the morphologies of the SW780 cells-derived exosomes were observed by TEM. **F** The uptake of PKH26-labeled SW780 cells-derived exosomes was examined by microscopy. Green: PKH26; Blue: DAPI. Scale bar:100 μm. **G** Exosome markers and negative control Calnexin and actin were detected by western blot. **H**, **I** Cell proliferation of HUVECs treated with SW780-Exos (**H**) or T24- Exos (**I**) was monitored by CCK-8 assay. **J** Angiogenesis, migration, and cell senescence of HUVECs were monitored by tube formation and transwell migration by β-gal staining assays with quantitative analysis (right panel), Scale bar:100 μm or 50 μm. Statistical analyses were performed using a two-tailed Student’s *t*-test, with significance levels indicated as *P < 0.05, **P < 0.01, and ****P < 0.001, representing significant differences between groups.

To explore the molecular intricacies underlying SLC7A7-mediated angiogenesis, we hypothesized that tumor cells with high SLC7A7 expression may influence the TME, thereby promoting angiogenic processes. To investigate this hypothesis, we harvested the supernatants from tumor cells with silenced SLC7A7. Using a chick chorioallantoic membrane (CAM) model, we validated that the conditioned medium (CM) from SLC7A7-knockdown tumor cells led to a significant reduction in the number and the bifurcation of vessels within the CAM (Fig. 4A, B). Indeed, the CM from SLC7A7 knockout cells exhibited substantial inhibition of angiogenesis, significantly reducing both the number of branching vessels and the overall length of new vessels (Fig. 4C, D). This observation prompted a comprehensive investigation into the identification of key components responsible for mediating angiogenesis in this context.

Considering the amino acid transport function of SLC7A7, we assessed the arginine and lysine content in the supernatant of stably transfected cells and observed no significant differences (Fig. S3A, B). Notably, exosomes are known to play a crucial role in promoting angiogenesis within the TME^25^. Therefore, we speculate that SLC7A7 may regulate angiogenesis through exosomal mechanisms.

Exosomes purified from SW780 or T24 cells-derived CM were identified as membrane vesicles of 100–150 nm by transmission electron microscopy (TEM) and nanoparticle tracking analysis (NTA) (Fig. 4E and S3C, D). PKH26-labeled SW780-derived exosomes (SW780-Exos) or T24-derived exosomes (T24-Exos) demonstrated successful uptake by HUVECs (Fig. 4F and S3E). Furthermore, these membrane vesicles tested positive for exosome markers CD63 and CD81 while showing negativity for the cell markers Calnexin and β-Actin, which served as negative controls (Fig. 4G).

To further investigate the role of SW780-Exos and T24-Exos in HUVECs, we assessed the proliferation of HUVECs treated with exosomes derived from SLC7A7 knockout cells (shSLC7A7-Exos) using a CCK-8 assay. As illustrated in Fig. 4H, I, shSLC7A7-Exos significantly reduced the proliferation of HUVECs when compared to shNC-Exosomes. Furthermore, tube formation assays clearly demonstrated that shSLC7A7-Exos diminished the formation of capillary-like structures by HUVECs (Fig. 4J and S3F). Additionally, transwell migration assays (Fig. 4J and S3G) and cell senescence assays (Fig. 4J and S3H) indicated that shSLC7A7-Exos reduced cell migration while promoting cell senescence in HUVECs, respectively. Collectively, these data suggest that SLC7A7 affects the proliferation, angiogenesis, migration, and senescence of HUVECS via cells-derived exosomes.

### SLC7A7 silencing-mediated upregulation of exosomal miR-152-3p facilitates angiogenesis in HUVECs

**Fig. 5:**
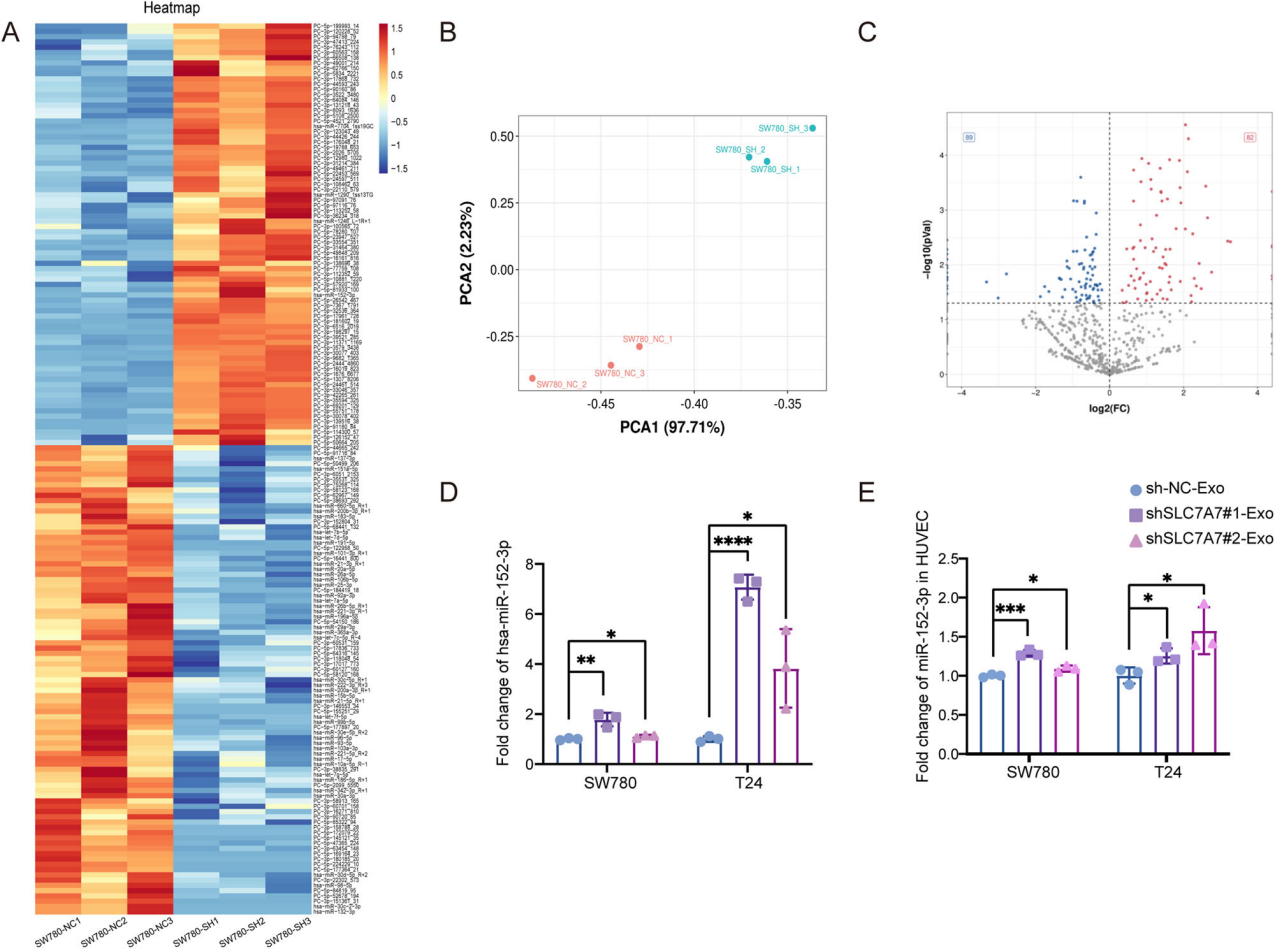
miR-152-3p is up-regulated in exosomes after silencing SLC7A7. **A** Heat map of the top 50 differential miRNA clusters in SW780 cells-derived exosomes. **B** PCA analysis of exosomes from SW780 cells with stably silenced SLC7A7. **C** Volcano map of differential miRNAs in SW780 cells-derived exosomes. **D** miR-152-3p level in SW780 or T24 cells-derived exosomes was detected by qRT-PCR. **E** miR-152-3p level in HUVECs was detected by qRT-PCR. Statistical analyses were performed using a two-tailed Student’s *t*-test, with significance levels indicated as *P < 0.05, **P < 0.01, ***P < 0.001, and ****P < 0.001, representing significant differences between groups.

Emerging evidence shows the correlation between exosomal miRNAs and angiogenesis in various cancers^26,27^. Therefore, we hypothesized that the involvement of miRNAs in SLC7A7-derived exosomes regulates angiogenesis. We next sequenced miRNAs from the isolated exosomes. As shown in Fig. 5A, B, qualitative analysis of the sequencing data revealed minimal intragroup variability within each exosome group and substantial differences between the two groups. This outcome validates the efficacy of the exosome separation process, confirms distinct compositional disparities between the groups, and fulfills essential criteria for sequencing. As illustrated in Fig. 5C, differential analysis of the miRNA-seq data identified 82 up-regulated miRNAs and 89 down-regulated miRNAs. Furthermore, by employing strict criteria of *P* < 0.05 and fold-change > 1.5, we selectively screened for the top 50 up-regulated miRNAs (Fig. 5A). Notably, among these is miR-152-3p, which has previously been validated to participate in regulating angiogenic pathways^28,29^. This compelling discovery suggests that SLC7A7-mediated exosomes may exert their angiogenic function, at least in part, through the modulation of miR-152-3p.

We next examined the level of exosomal miR-152-3p in SW780-Exos and T24-Exos, along with the levels in exosome-treated HUVECs by qRT-PCR. Compared with control, miR-152-3p was dramatically increased in exosomes purified from the CM of SLC7A7 knockout cells (Fig. 5D). Moreover, miR-152-3p level in HUVECs treated with shSLC7A7-Exos was significantly higher than that in shNC-Exo group (Fig. 5E). These data suggest that miR-152-3p is increased in exosomes derived from SLC7A7 knockout cells and associated with angiogenesis.

### Exosomal miR-152-3p inhibited the proliferation, invasion, and angiogenesis of HUVECs

**Fig. 6:**
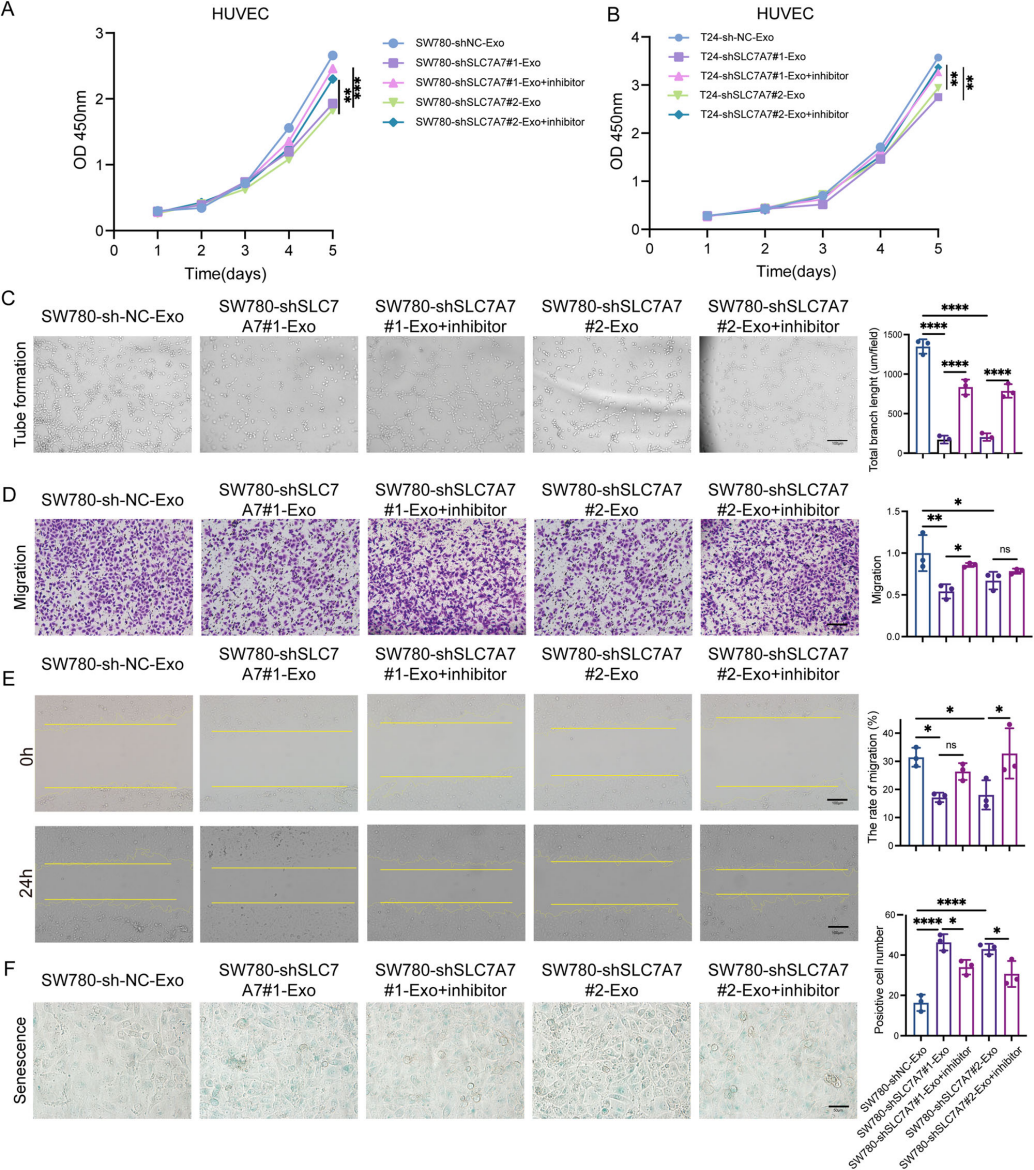
Exosome-derived miR-152-3p inhibited the proliferation, migration, and metastasis of HUVECs. **A**, **B** Cell proliferation of HUVECs was monitored by CCK-8 assay. **C** Angiogenesis of HUVECs was assessed by tube formation assay. Scale bar:100 μm. **D**, **E** Migration of HUVECs was monitored by transwell migration (**D**) and wound healing assays (**E**) with quantitative analysis (right panel). Scale bar:100 μm. **F** Cell senescence was detested by β-gal staining with quantitative analysis (right panel). Scale bar: 50 μm. Statistical analyses were performed using a two-tailed Student’s *t*-test, with significance levels indicated as **P < 0.01 and ***P < 0.001, representing significant differences between groups.

To further investigate the role of exosomal miR-152-3p in HUVECs, these cells were transfected with an miR-152-3p inhibitor following treatment with exosomes. As shown in Fig. 6A, B, the miR-152-3p inhibitor markedly enhanced the proliferation of HUVECs in a time-dependent manner compared to treatment with shSLC7A7-Exos. Moreover, tube formation assays clearly indicated that the miR-152-3p inhibitor encouraged the development of capillary-like structures in HUVECs (Fig. 6C and S4A). Additionally, both transwell migration (Fig. 6D and S4B) and wound healing assays (Fig. 6E and S4C) demonstrated that the miR-152-3p inhibitor promoted the migration of HUVECs. In contrast, the miR-152-3p inhibitor diminished cell senescence in HUVECs (Fig. 6F). Together, these results provide strong evidence that SLC7A7 plays a critical role in angiogenesis by regulating the levels of exosomal miR-152-3p.

### MiR-152-3p inhibits angiogenesis by targeting FGFR3

**Fig. 7:**
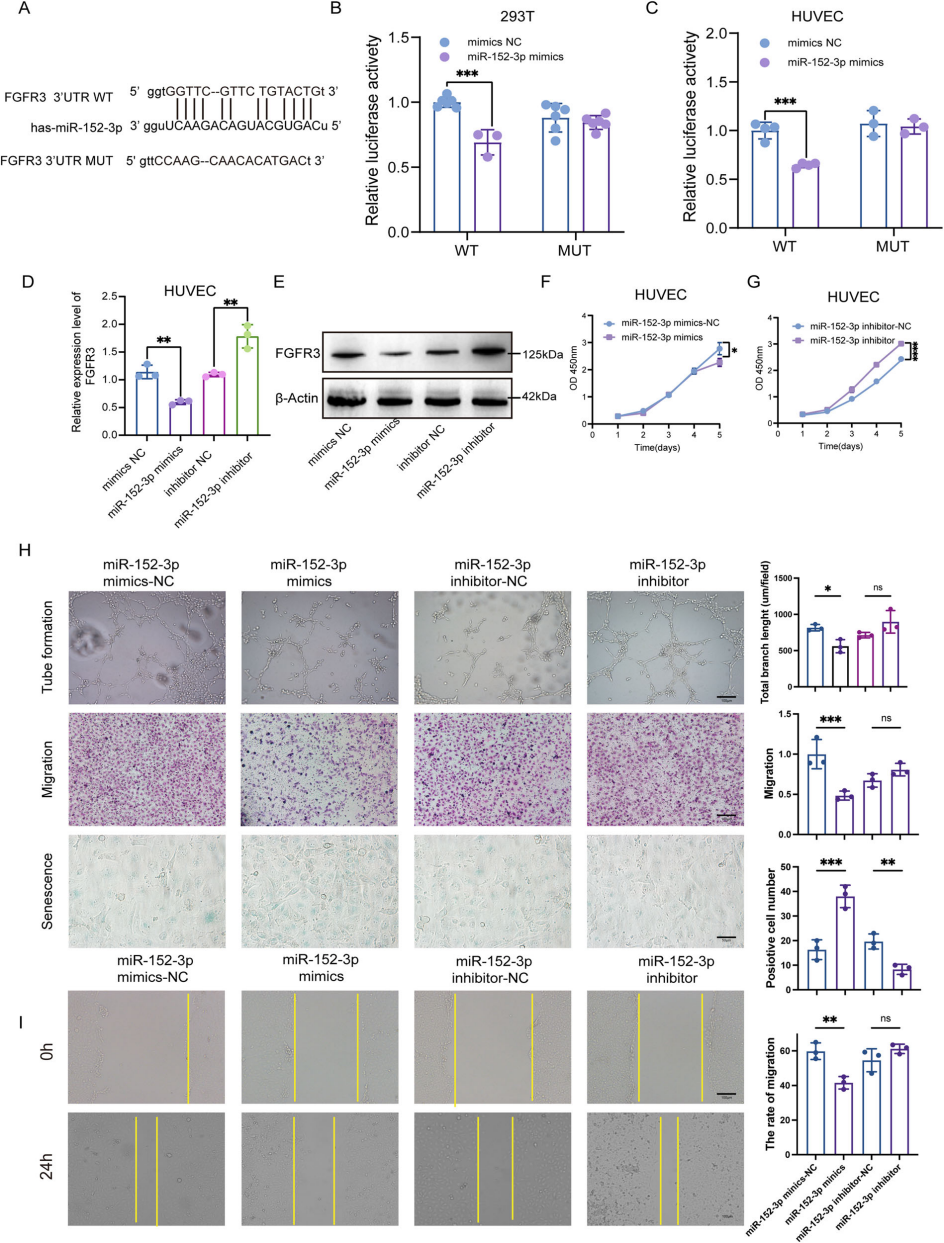
miR-152-3p inhibits angiogenesis by targeting FGFR3. **A** Putative binding site between FGFR3 3’UTR and miR-152-3p. **B**, **C** Relative luciferase activity was measured by dual-luciferase reporter assay in 293T (**B**) and HUVECs (**C**). **D**, **E** The mRNA (**D**) and protein levels (**E**) of FGFR3 were determined by qRT-PCR and western blot, respectively. **F**, **G** Cell proliferation of HUVECs was monitored by CCK-8 assay. **H** Angiogenesis, migration, and cell senescence of HUVECs were assessed by tube formation assay, transwell migration assays, and β-gal staining, respectively, with quantitative analysis (right panel). Scale bar:100 μm or 50 μm. **I** Cell migration was assessed by wound healing assay with quantitative analysis (right panel). Scale bar:100 μm. Statistical analyses were performed using a two-tailed Student’s *t*-test, with significance levels indicated as *P < 0.05, **P < 0.01, ***P < 0.001, and ****P < 0.001, representing significant differences between groups.

Indeed, miRNAs function by binding to the 3’ UTRs of their target mRNAs. In our investigation, we utilized Starbase to predict potential targets of miR-152-3p and identified FGFR3 as a likely candidate. Figure 7A visually shows the binding sites of miR-152-3p to FGFR3. To verify this interaction in experiments, we performed dual-luciferase reporter analysis in both 293 T cells and HUVECs. Fragments of FGFR3’s 3’ UTR, encompassing either the wild-type (WT) or mutant (Mut) miR-152-3p binding sites, were cloned into pmiR-GLO (pmiR-GLO-NRF23’ UTR WT or Mut). Notably, luciferase activity was significantly reduced in cells transfected with pPMIR-GLO-Nrf23 ‘UTR WT and miR-152-3p mimics compared with cells transfected with PPMIR-GLO-Nrf23’ UTR WT and negative control mimics (Fig. 7B, C). Conversely, transfection of PMIRGLO-NRF23’ UTR MUT, together with miR-152-3p mimics, did not elicit any effect on luciferase activity (Fig. 7B, C).

To delve further into the impact of miR-152-3p on FGFR3 expression, we transfected miR-152-3p mimics or NC mimics into HUVECs. Both qRT-PCR and Western blotting consistently demonstrated that miR-152-3p overexpression led to a reduction in FGFR3 mRNA and protein levels (Fig. 7D, E). Additionally, the overexpression of miR-152-3p hindered the proliferation (Fig. 7F, G), tubule formation (Fig. 7H), and migration (Fig. 7H, I) of HUVECs while promoting cellular senescence (Fig. 7H). Conversely, transfection with miR-152-3p inhibitors yielded the opposite result (Fig. 7F–I). These findings collectively suggest that miR-152-3p inhibits FGFR3 expression by specifically targeting the 3’ UTR of FGFR3. Consequently, this regulatory interaction hampers the migratory invasion, tubule formation, and overall angiogenic potential of HUVECs.

### SLC7A7 facilitates angiogenesis through miR-152-3p/FGFR3 axis

We subsequently investigated whether SLC7A7 facilitates angiogenesis via the miR-152-3p/FGFR3 axis in vivo. We subcutaneously injected either T24-shControl or T24-shSLC7A7-silenced cells into nude mice. Compared to the vehicle control, SLC7A7 knockdown significantly delayed tumor volume and weight (Fig. S5A–D) and increased the expression of miR-152-3p (Fig. S5E), which consequently resulted in a decrease in CD32 and FGFR3 levels (Fig. S5F-I). These findings suggest that the inhibition of SLC7A7 disrupts angiogenesis through the miR-152-3p/FGFR3 signaling network within the TME.

## Discussion

This study showed that SLC7A7 is elevated in BCa tissue and that upregulation of SLC7A7 is positively correlated with angiogenesis in BCa patients. Exosomal miR-152-3p derived from SLC7A7-silenced BCa cell exerted blocking angiogenesis roles in vitro and in vivo. Mechanistic studies further revealed that the exosomal miR-152-3p/FGFR3 axis was implicated in angiogenesis and tumor progression in vitro and in vivo. Our findings reveal the biological role of exosome miR-152-3p in BCa and lay the foundation for the clinical application of FGFR3 targeted inhibition therapy.

The prognosis for patients with advanced BCa is notably bleak, and the underlying mechanisms of disease progression are intricate. Our bioinformatics analysis of the GEO dataset GSE32548 identified differentially expressed genes enriched in protein absorption and degradation processes (Fig. S1), suggesting a link to amino acid metabolism. Among these, SLC7A family genes were notably prominent (Fig. 1B), with SLC7A7 emerging as the most distinctive gene (Fig. 1C). SLC7A7 is highly expressed in a variety of cancers and could be a predictor of poor prognosis. In our analysis of clinical BCa samples, both protein and mRNA levels of SLC7A7 were significantly elevated, with this upregulation closely associated with muscular invasion (Fig. 1D–J), suggesting a pro-cancer role in BCa progression. GSEA further revealed a strong association between SLC7A7 and angiogenesis (Fig. 2A–E).

To further elucidate the mechanisms through which SLC7A7 promotes angiogenesis, we hypothesized that the TME facilitates communication between BCa cells and endothelial cells. The SLC7A7 gene encodes the YLAT1 light chain of the Y + L system, which, in conjunction with SLC3A2, mediates transmembrane amino acid transport^9,30,31^. Notably, cationic amino acids such as arginine and lysine are primarily extruded from cells through membranes in a sodium-dependent manner. Functional mutations in SLC7A7 can lead to ion transport dysfunction and are associated with various clinical symptoms^9^. For instance, elevated SLC7A7 expression is linked to poor prognosis in Glioblastoma multiform^32^. While in chemotherapy-resistant ovarian cancer, it regulates drug influx/efflux, influencing chemotherapy responses^33^. In non-small-cell lung carcinoma, SLC7A7 expression positively correlates with the level of immune infiltration^10^. Studies have shown that overexpression of SLC7A7 can decrease the proportion of apoptotic Jurkat cells, increase the proportion of G1 phase cells, and enhance the migration and invasion of tumor cells^34^. However, the specific role of SLC7A7 in BCa remains unclear. In our investigation, we treated HUVECs with supernatants from SLC7A7 silencing-BCa cell media. Interestingly, we found that supernatants following SLC7A7 silencing did not significantly promote angiogenesis (Fig. 4A–C). Furthermore, no significant difference was found in the concentration of arginine in the supernatant (Fig. S3A-B). These results suggest that SLC7A7 may not promote angiogenesis by affecting arginine content.

Research has consistently demonstrated the pivotal role of exosomes in the regulation of the TME, particularly in the context of angiogenesis^25^. Exosomes released by tumor cells within the angiogenesis pathway encompass various angiogenic factor proteins and regulatory nucleic acid molecules^35^. These exosomes are easily engulfed by endothelial cells in the TME, which has an important effect on promoting the growth and migration of endothelial cells^36,37^. To further investigate the effect of SLC7A7 on angiogenesis, exosomes were isolated from the supernatant of BCa cells silenced with SLC7A7 by overspeed centrifugation. Treatment of HUVECs with these exosomes revealed a significant inhibition of angiogenesis (Fig. 4H–J, S3F-H). Subsequently, by miRNA sequencing, we screened for up-regulated miRNAs with at least a 50-fold difference (Fig. 5A). Notably, miR-152-3p, previously implicated in angiogenesis^28,29^, emerged as a prominent candidate.

To further delineate the mechanism by which exosomal miR-152-3p regulated angiogenesis in BCa, mechanistic studies were conducted. We engineered vectors for miR-152-3p mimics, miR-152-3p inhibitor, and their respective controls, subsequently transfected into HUVECs. Remarkably, the miR-152-3p inhibitor was found to reverse the inhibitory effects observed with SLC7A7-silenced exosomes on angiogenesis, as well as the migration and tube formation of HUVECs (Fig. 6 and Fig. S4). Further investigations led us to predict FGFR3 as a potential target protein of miR-152-3p through database analysis. We then verified this prediction with dual-luciferase reports, WB, and QPCR (Fig. 7B–E). Additionally, transfection of miR-152-3p mimics significantly attenuated the ability of HUVECs to undergo tube formation, migration, and invasion, while inducing cell senescence (Fig. 7F–I). Conversely, transfection of the miR-152-3p inhibitor significantly enhanced tube formation, migration, and invasion capabilities of HUVECs, while alleviating cell senescence (Fig. 7F–I). These findings collectively underscore the pivotal role of miR-152-3p in modulating angiogenesis and associated cellular processes, further supporting its potential as a key therapeutic target in the context of BCa.

There are several limitations to our study. Firstly, although we have confirmed that miR-152-3p is a potential mediator, we did not explore the specific mechanism of how SLC7A7 regulates miR-152-3p levels. Exosomes are 40-160 nm vesicles generated during plasma membrane double invagination and intracellular multivesicular body (MVB) formation that contain intraluminal vesicles^38^. Moreover, exosomes can consist of proteins, lipids, miRNAs, and other RNA species; their heterogeneity can lead to different exosome content^39^. However, there is currently no report on whether SLC7A7 affects the production or heterogeneity of exosomes. Further investigation is warranted to test this. Additionally, mutations in SLC7A7 causing cation transporter dysfunction have been extensively studied^40^; however, the levels of arginine and lysine in cells stably silencing SLC7A7 in our study were as high as those in NC-cells. Therefore, it is necessary to follow up with the metabonomics study to explore the changes in intracellular and extracellular contents. Finally, we found promise in targeted FGFR3 therapy in BCa; however, further investigations are necessary to verify whether targeted FGFR3 therapy is effective in animal studies or clinical patients.

**Fig. 8:**
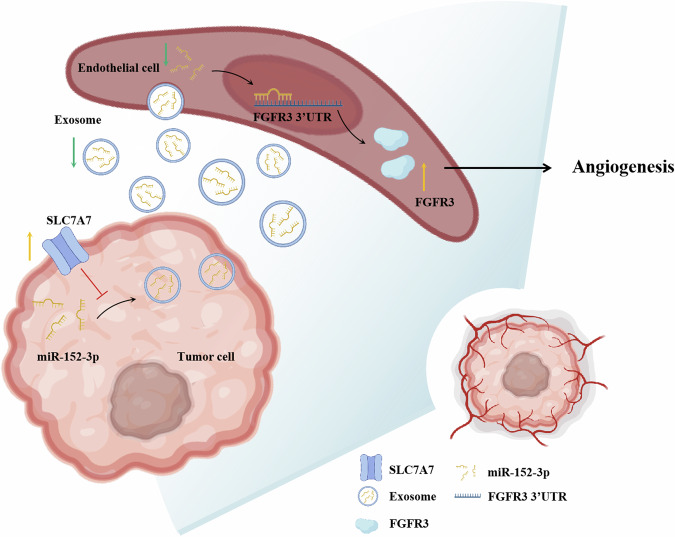
Schematic of SLC7A7 promoted angiogenesis via exosomes-mediated miR-152-3p /FGFR3.

In summary, our findings substantiate that SLC7A7 inhibits the secretion of exosome-derived miR-152-3p from BCa cells, which in turn attenuates the inhibitory effect on FGFR3 in HUVECs, thereby effectively promoting angiogenesis (Fig. 8). Consequently, the level of exosomal miR-152-3p emerges as a promising prognostic biomarker, holding potential clinical significance. Furthermore, our study suggests that targeting FGFR3 through anti-angiogenic therapy could open up new prospects for the treatment of BCa. The intricate interactions between SLC7A7, exosome-mediated miR-152-3p, and FGFR3 provide valuable insights into potential therapeutic strategies, paving the way for more effective and targeted interventions in the treatment of BCa.

## Methods

### Collection of clinical specimens

A cohort comprising 30 paired BCa tissues and adjacent non-tumor bladder mucosal tissues was procured from patients diagnosed with BCa who underwent radical cystectomy at the Department of Urology, Nanfang Hospital, Southern Medical University, spanning from September 2020 to November 2022. This study was approved by the Ethics Committee of the Southern Hospital and was conducted Declaration of Helsinki, with all patients participating in the study providing informed consent.

### Cell culture

Human BCa cell lines, including T24, UMUC-3, SW780, human bladder epithelial cells SV-HUC-1, and Human umbilical vein endothelial cells (HUVECs), were sourced from the Stem Cell Bank, Chinese Academy of Sciences in Shanghai. Authentication of all cell lines was recently conducted. The cell lines were cultured using Dulbecco’s Modified Eagle’s medium (DMEM) (Gibco) with 10% fetal bovine serum (FBS) and 1% penicillin/streptomycin for T24, SW780, HUVECs, UMUC3 cells. And HUVECs were cultured with 1% endothelial growth factor. SV-HUC-1 was cultured in RPMI 1640 medium (Gibco) supplemented with 10% FBS and 1% penicillin/streptomycin. All cells were cultured in a humidified incubator at 37 °C with 5% CO2.

### Bioinformatics analysis

Download BCa patient data from both the TCGA and GEO databases and stratify the samples into two distinct groups: NMIBC and MIBC. Subsequently, conduct a transcriptome differential expression analysis (DEGs) with specific criteria (LogFC > 2 and FDR < 0.05). Following the identification of differentially expressed genes, perform Gene Ontology (GO) and KEGG enrichment analyses on these genes. Notably, the analysis reveals an upregulation of SLC7A7 in MIBC, providing valuable insights into its potential role in the progression of MIBCa.

### Protein extraction and Western blot

Proteins from cells and tissues were extracted using RIPA lysis buffer (FUDE BIOLOGICAL, Hangzhou, China) supplemented with protease and phosphatase inhibitors (FUDE BIOLOGICAL). The lysate was treated with SDS-PAGE protein buffer (FUDE BIOLOGICAL) and denatured at 100 °C in a water bath for 5 min. Following separation by SDS-polyacrylamide gel electrophoresis, the protein samples were transferred onto a polyvinylidene fluoride (PVDF) membrane (Millipore). The membrane was then blocked with 5% quick-blocking buffer (FUDE BIOLOGICAL) for 20 min, followed by overnight incubation with the primary antibody at 4 °C. Subsequently, the membrane underwent a 2-hour incubation with the secondary antibody (SA00001-2 and SA00001-1, roteinTech). β-actin immunoblotting (66009-1-Ig, ProteinTech) was employed for the normalization of protein loading. All immunoblots presented in corresponding figure panels were derived from identical experimental conditions and processed simultaneously during electrophoresis and detection procedures. The Western blot images were captured using the Tanon-4600 imaging system (Biotanon). Complete unprocessed membrane images are available in Supplementary Fig. 6. The following primary antibodies were used: anti-SLC7A7 (PHD3349, Abmart), anti-FGFR3 (A19052, Abclonal), anti-CD31 (28083-1-AP, ProteinTech), anti-CD63 (32151-1-AP, ProteinTech), anti-CD81 (27855-1-AP, ProteinTech), anti-Calnexin (10427-2-AP, ProteinTech).

### Transfection and establishment of stable clone cells, cell transfection

T24 cells and SW780 cells were subjected to stable transfection with SLC7A7 lentivirus, resulting in T24-shSLC7A7 and SW780-shSLC7A7 cell lines, respectively. Additionally, empty vector control sublines were established as T24-shNC and SW780-shNC. The transient transfection of HUVECs was performed using Lipofectamine 3000 (Invitrogen) with chemically synthesized miR-152-3p mimics, inhibitors, or negative controls at a concentration of 20 mM.

### Subcutaneous xenograft model

The animal experiments received support from the Animal Experiment Ethics Committee of Nanfang Hospital, Southern Medical University. All the BALB/c nude mice, aged 4-5 weeks, were randomly divided into three groups: Group 1 received T24-shNC cells (control group), Group 2 received T24-shSLC7A7#1 cells, and Group 3 received T24-shSLC7A7#2 cells. Stable T24-shSLC7A7#1, T24-shSLC7A7#2, and control T24sh-NC cells in the logarithmic growth phase were selected. A single-cell suspension was prepared with a cell density of 1 × 10^7^/ml.

The nude mice were housed in a specific pathogen-free (SPF) animal room, and their growth, activity, body weight, and changes in implanted tumor volume were regularly monitored and measured. After four weeks, the nude mice were euthanized under anesthesia, and the subcutaneous tumor tissue was extracted, collected, weighed, and photographed for further analysis. The maximum tumor size/burden did not exceed the Animal Experiment Ethics Committee requirement. The experimental procedures were conducted in accordance with the National Laboratory Animal Care and Use Research Committee guidelines.

### Real-time PCR

Total RNA from cells and tissue specimens was extracted using TRIzol following the manufacturer’s instructions. For cDNA synthesis, 1 µg of RNA per sample was utilized with a TaqMan reverse transcription reagent kit (Applied Biosystems). Quantitative real-time PCR was conducted on a 7500 real-time PCR system (Applied Biosystems) using a SYBR Premix Ex Taq kit for real-time PCR.

### IHC staining

Paraffin-embedded tumor and tissues were deparaffinized and rehydrated as previously described^41^. Following antigen retrieval and blocking, sections were incubated overnight at 4 °C with anti-CD31 (1:50, ab182981, Abcam) and anti-SLC7A7 (1:200, ab180630, Abcam) antibodies, respectively. Subsequently, the sections were subjected to incubation with the appropriate secondary antibodies. The immunoreactivity was visualized using the DAB substrate (Thermo Fisher Scientific).

### Extraction of exosomes from the cell culture supernatant

The designated BCa cells at 80% confluence were cultured in serum-free DMEM. After three days, the CM was collected, filtered, and stored at −80 °C. Exosomes were isolated from the CM using a differential centrifugation method. Specifically, the CM was subjected to sequential centrifugation steps at 300 × *g* for 30 min, 3000 × *g* for 30 min, 20,000 × *g* for 30 min, and 100,000 × *g* for 80 min, all performed at 4 °C. The resulting pellets were washed twice with PBS and further purified by centrifugation at 100,000 × *g* for 80 min at 4 °C.

### TME and NTA

For the identification of exosomes from BCa cells, 20 μl of PBS suspension was applied to a copper grid and air-dried for 5 min at room temperature. Any residual liquid was gently removed using filter paper on both sides. Subsequently, 20 μl of 3% phosphotungstic acid was added, allowing for negative staining for 5 min at room temperature. After staining, the excess phosphotungstic acid solution was carefully removed, and the copper grid was subjected to heat under a 65 °C incandescent lamp for approximately 15 min. The grid was air-dried at room temperature for an additional 5 min. Following negative staining, the copper grid was examined and photographed using a transmission electron microscope. NTA (N30E, NanoFCM) was used to analyze the size and concentration of exosomes.

### Identification of exosome marker proteins

The cell supernatant of BCa tumor cell culture collected as described above was used to extract exosomes by ultracentrifugation. 100 μg of each exosome was placed on ice, added with 100 μl of high-strength RIPA, and sonicated for 15 min to completely dissolve the exosomes. The mixture was then mixed on ice and left at 4 °C for 30 min. The exosomes were centrifuged at 12,000 rpm for 30 min in a pre-cooled centrifuge. Then the exosome marker proteins CD63 and CD81 were detected by Western blot.

### Exosome uptake assay

Exosome uptake assay was performed using PKH67 Kit (Sigma-Aldrich) following the manufacturer’s instructions. PKH67-labeled exosomes (10 μg) were resuspended in 100 μl of PBS and incubated with HUVECs at a concentration of 1 × 10^5^ cells. After 24 h, cells were harvested for immunofluorescence analysis. The images were acquired using an Olympus confocal microscope (Olympus).

### Tube formation assay

In vitro angiogenesis was assessed by tube formation assay as previously described^42^. Briefly, HUVECs were seeded onto Matrigel (Corning)-coated 96-well plates (1 × 10^5^) and treated with indicated exosomes derived from BCa cells. After 6 h, tube was captured using a digital camera system (Olympus), and the total length and number of branches of the tubes were analyzed using Image J software.

### Cell counting kit-8 assay

Cell viability was assessed using the Cell Counting Kit-8 assay, following the manufacturer’s instructions (Dojindo). Cells were seeded in 96-well plates at a density of 1000 cells per well. Incubation continued for 0, 24, 48, 72 and 96 h after transfection. Subsequently, 10 μl of CCK-8 solution was added to each well and incubated for 1.5 h at 37 °C. The absorbance at 450 nm was then measured using a spectrometer (Thermo Fisher Scientific).

### Wound healing assay

Transfected cells were plated into 6-well plates. Wounds were manufactured by a 200 μl pipet tip. PBS was used to wash the cell debris three times, after which the cells were incubated with serum-free media. The wound was permitted to heal for 24 h. Cells were photographed at 0 h and 24 h using a phase-contrast microscope.

### Cell migration assay

In vitro migration was evaluated using the Transwell assays. HUVECs (2 × 10^4^) were first resuspended in 200 μl serum-free DMEM medium and then seeded into a Transwell chamber. Then,1 ml culture medium was added to the lower chamber. HUVECs were allowed to migrate for 24 h at 37 °C in a humidified atmosphere of 5% CO2. After permeabilized with methanol and staining with 0.1% crystal violet, the migrated cells were counted using a light microscope.

### CAM assay

Fertilized eggs obtained from South China Agricultural University were incubated at 37.5 °C with 60% humidity for 6 days, with the removal of unqualified eggs. A window was opened at the air chamber of the egg, and the inner shell membrane was removed. A sterilized rubber ring with a diameter of 1 cm was placed on the CAM, and 100 µl of serum-free medium or prepared CM was dropped into the ring. The eggs were then incubated further at 37 °C with 60% humidity for 72 h. After this period, angiogenesis within the ring was photographed using a digital camera. The number of neovascularizations originating from the CAM blood vessel was counted. The ratio of vascular density was calculated as the number of new blood vessels after CM treatment divided by the number of new blood vessels treated with a serum-free medium. Each treatment group utilized *n* = 3 eggs for analysis.

### Statistical analysis

All data are presented as the mean ± SD from at least three independent experiments. The statistical differences between the two groups were calculated using a Student’s *t-*test (two-tailed; unpaired) by GraphPad Prism 8 software, with *P* < 0.05 considered significant. Survival analysis was performed by using the Kaplan–Meier method. Asterisks generally indicate: *P < 0.05, **P < 0.01, ***P < 0.001 and ****P < 0.0001, n.s. = not significant. Additional method descriptions can be found as supplementary information.

## Supplementary information

Supplementary Materials

## Data Availability

The miRNA-seq data that supporting this finding of this study could be obtained from the NCBI Sequence Read Archive (https://www.ncbi.nlm.nih.gov/) with accession code PRJNA123456.
