# Supplementary material for: Exosomes containing miR-152-3p targeting FGFR3 mediate SLC7A7-induced angiogenesis in bladder cancer
Source: NPJ Precis Oncol. 2025 Mar 12;9:71. doi: 10.1038/s41698-025-00859-z (PMC11903784; doi:10.1038/s41698-025-00859-z)
Supplement: Supplementary file 1 — Supplementary Materials [file 41698_2025_859_MOESM1_ESM.docx]

**Supplementary Information**

**Exosomes containing miR-152-3p targeting FGFR3 mediate SLC7A7-induced angiogenesis in bladder cancer**

Chun Cao, Yu Wang , Xiaolin Deng, Xinlei Zhao, Yuwen Chen, Wanlong Tan, Fan Deng and Fei Li

Contents

[Supplementary Figures 2](#_Toc181088467)

[Supplementary Tables 11](#_Toc181088468)

# Supplementary Figures

**Supplementary Figure 1**


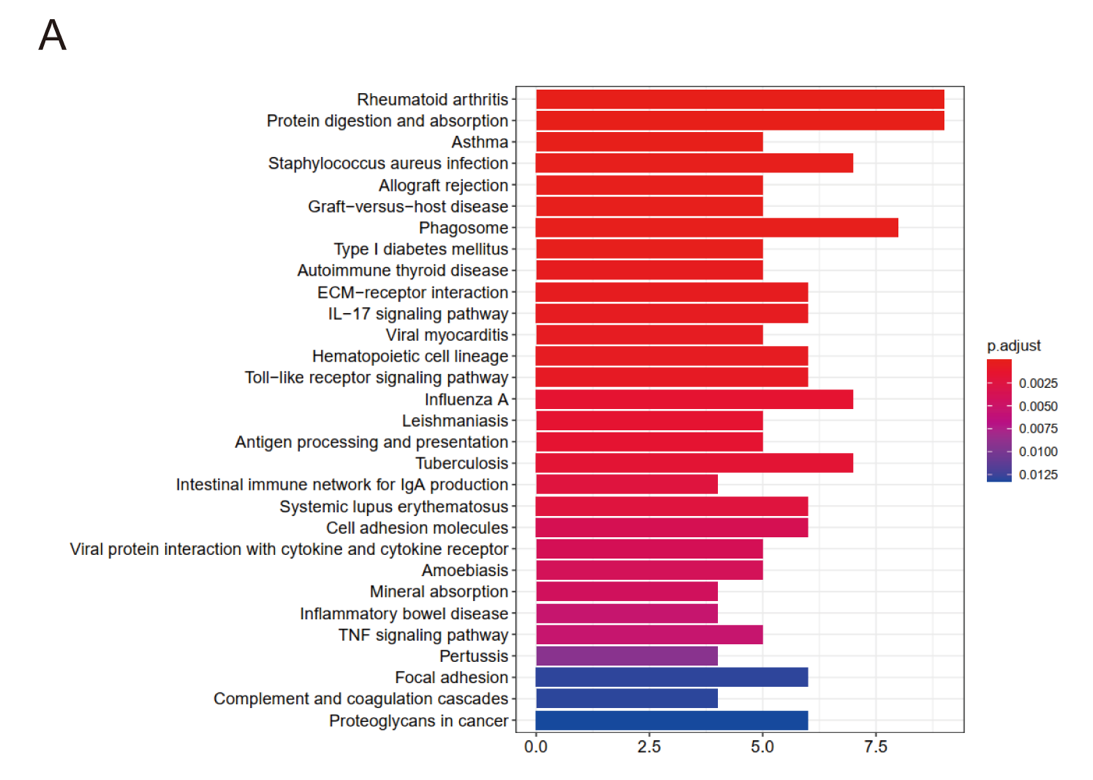


**Supplementary Figure 1. SLC7A7 in MIBCa high expression and is associated with poor prognosis**

1. KEGG enrichment analysis of upregulated genes in MIBCa.

**Supplementary Figure 2**


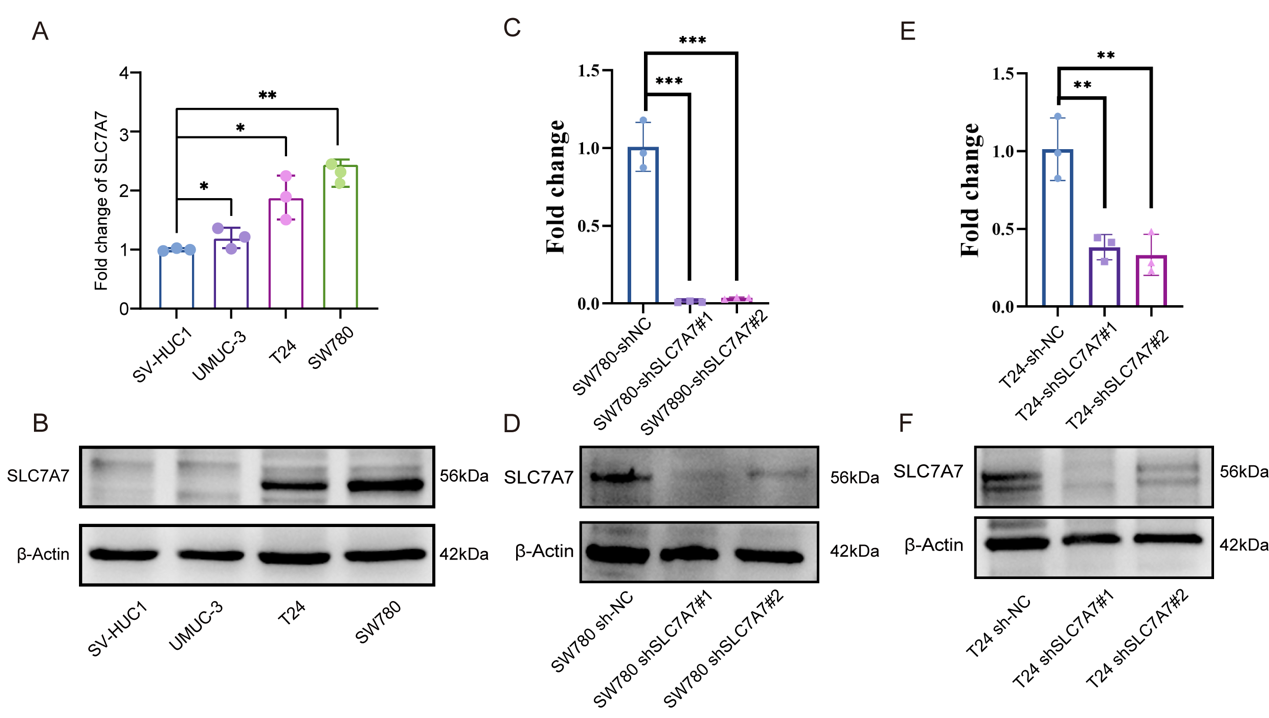
**Supplementary Figure 2. SLC7A7 promotes HUVECs angiogenesis *in vivo***

(A-B) The mRNA (A) and protein expression (B) of SLC7A7 in bladder cancer cells (UMUC-3, T24 and SW780) and uroepithelial cells (SV-HUC-1) were measured by qRT-PCR and western blot respectively. (C-F) The mRNA (C and E) and protein expression (D and F) of SLC7A7 in SW780 or T24 cells were measured by qRT-PCR and western blot respectively. Statistical analyses were performed using a two-tailed Student’s t-test, with significance levels indicated as *P < 0.05, **P < 0.01, ***P < 0.001, and ****P < 0.001, representing significant differences between groups.

**Supplementary Figure 3**


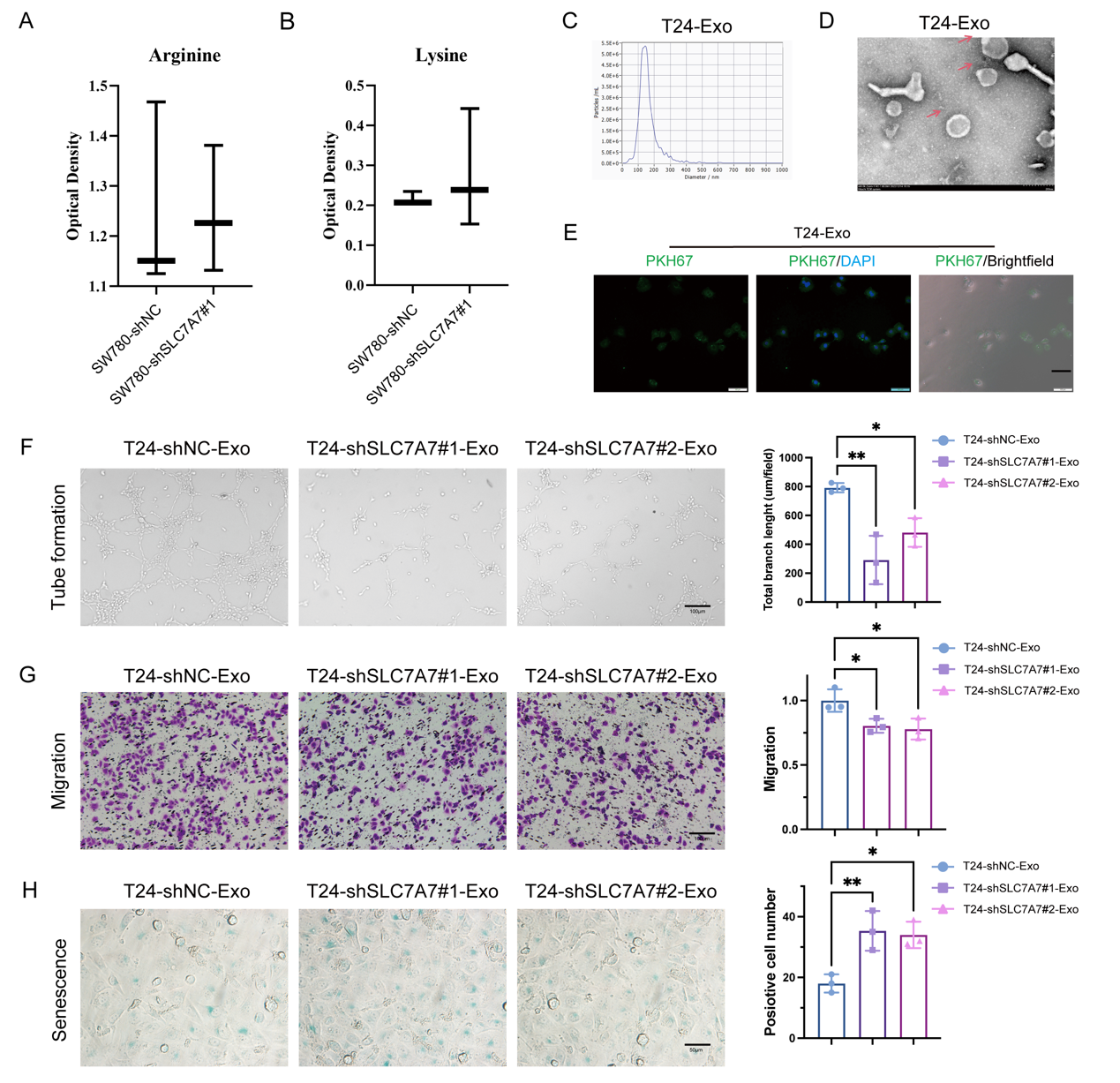
**Supplementary Figure 3. SLC7A7 promotes BCa angiogenesis through exosomes.**

(A-B) The levels of arginine and lysine in the supernatant of SW780 cells. (C) The size of the exosomes were analyzed by NTA. (D)The morphologies of the T24 cells-derived exosomes were observed by TEM. (E) The uptake of PKH26-labeled T24 cells-derived exosomes was examined by microscopy. Green: PKH26; Blue: DAPI. Scale bar:100 μm. (F) Angiogenesis of HUVECs was assessed by tube formation assay with quantitative analysis (right panel). Scale bar:100 μm. (G) Migration of HUVECs was monitored by transwell migration assay with quantitative analysis (right panel). Scale bar:100 μm. (H) Cell senescence of HUVECs were detested by β-gal staining with quantitative analysis (right panel). Scale bar: 50 μm. Statistical analyses were performed using a two-tailed Student’s t-test, with significance levels indicated as *P < 0.05, **P < 0.01 and ***P < 0.001, , representing significant differences between groups.

**Supplementary Figure 4**


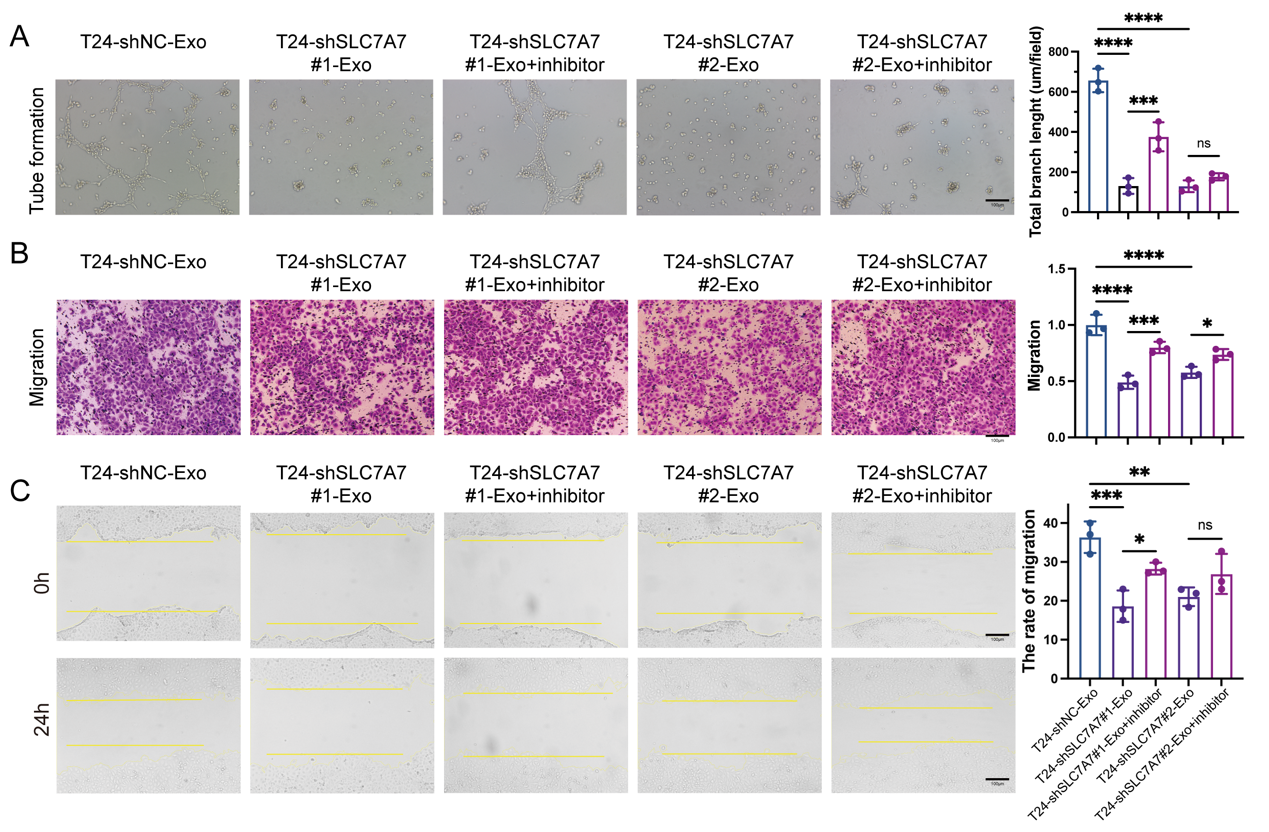
**Supplementary Figure 4. Exosome-derived miR-152-3p inhibited the proliferation, invasion and metastasis of HUVECs**

(A) Angiogenesis of HUVECs was assessed by tube formation assay with quantitative analysis (right panel). Scale bar:100 μm. (B) Migration of HUVECs was monitored by transwell migration assay with quantitative analysis (right panel). Scale bar:100 μm. (C) Cell migration was assessed by wound healing assay with quantitative analysis (right panel). Scale bar:100 μm. Statistical analyses were performed using a two-tailed Student’s t-test, with significance levels indicated as *P < 0.05, **P < 0.01, ***P < 0.001, and ****P < 0.001, representing significant differences between groups.

**Supplementary Figure 5**


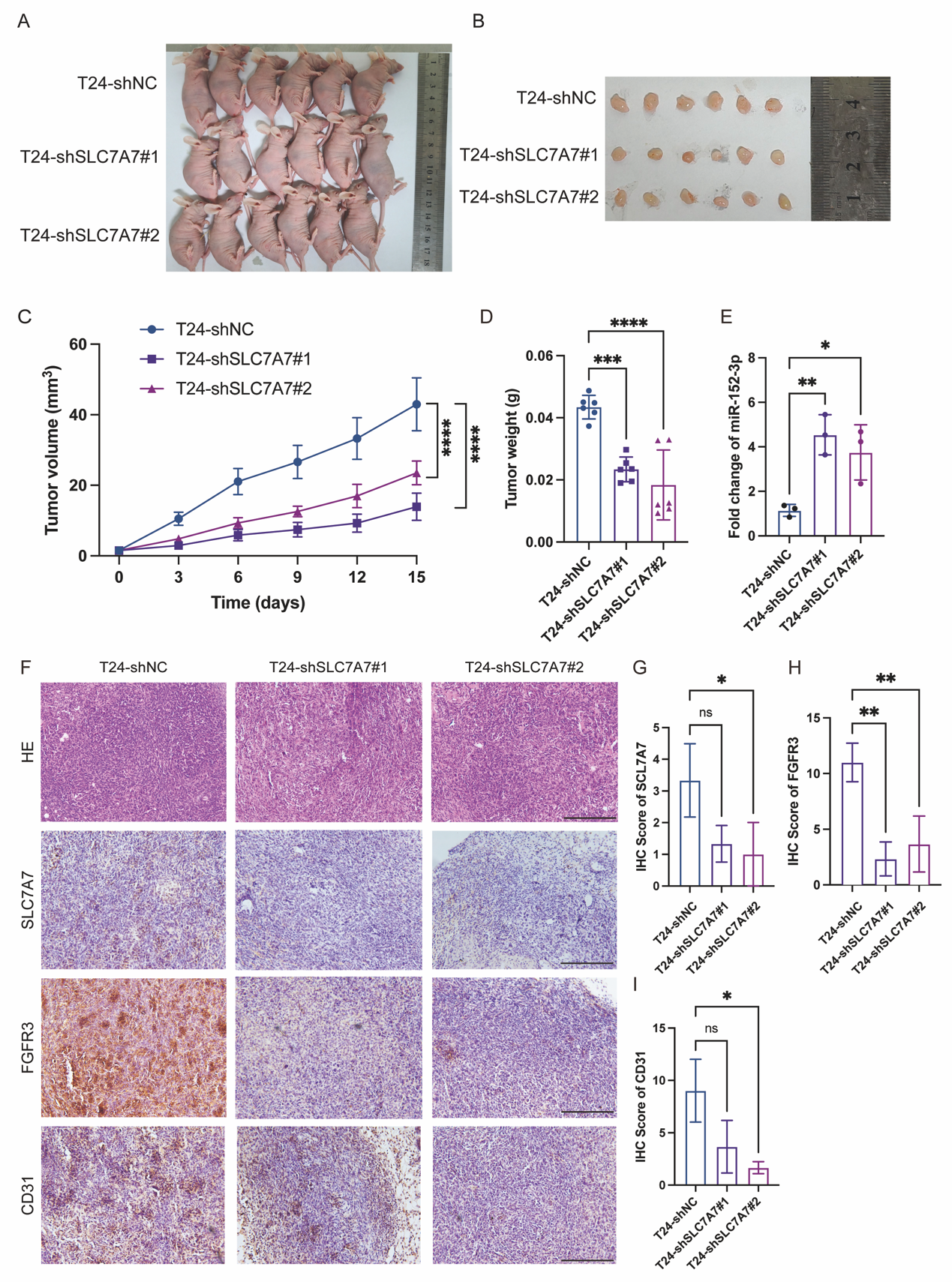


**Supplementary Figure 5. SLC7A7 facilitates angiogenesis through miR-152-3p/FGFR3 axis**

(A-D) Subcutaneous transplantation tumor model imaging (A) and tumor images (B) of the indicated treated mouse from six mouse each group. The tumor volume was measured regularly (C). The mice were sacrificed at appropriate times to remove the tumors and weigh the tumors (D). (E) miR-152-3p level in tumor issue was detected by qRT-PCR. (F-I) Representative photographs of tumor morphology with HE staining. The expression of SLC7A7, CD31and FGFR3 in tumor tissues of nude mice was checked by IHC with quantitative analysis (G-I). Scale bar:100 μm. Statistical analyses were performed using a two-tailed Student’s t-test, with significance levels indicated as *P < 0.05, **P < 0.01, ***P < 0.001, and ****P < 0.001, representing significant differences between groups.

**Supplementary Figure 6**

**Uncropped images of the immunoblots**

**
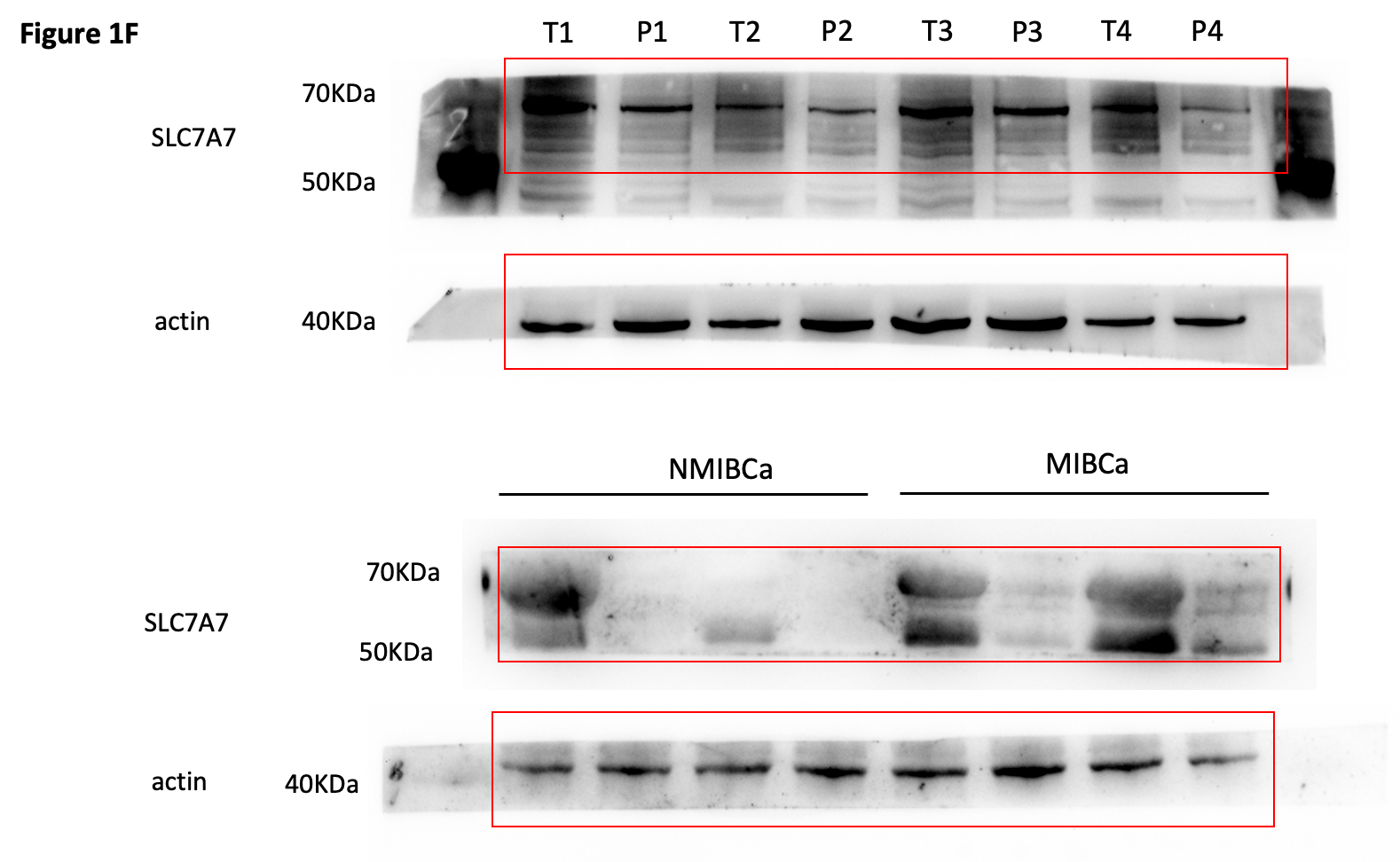
**

**
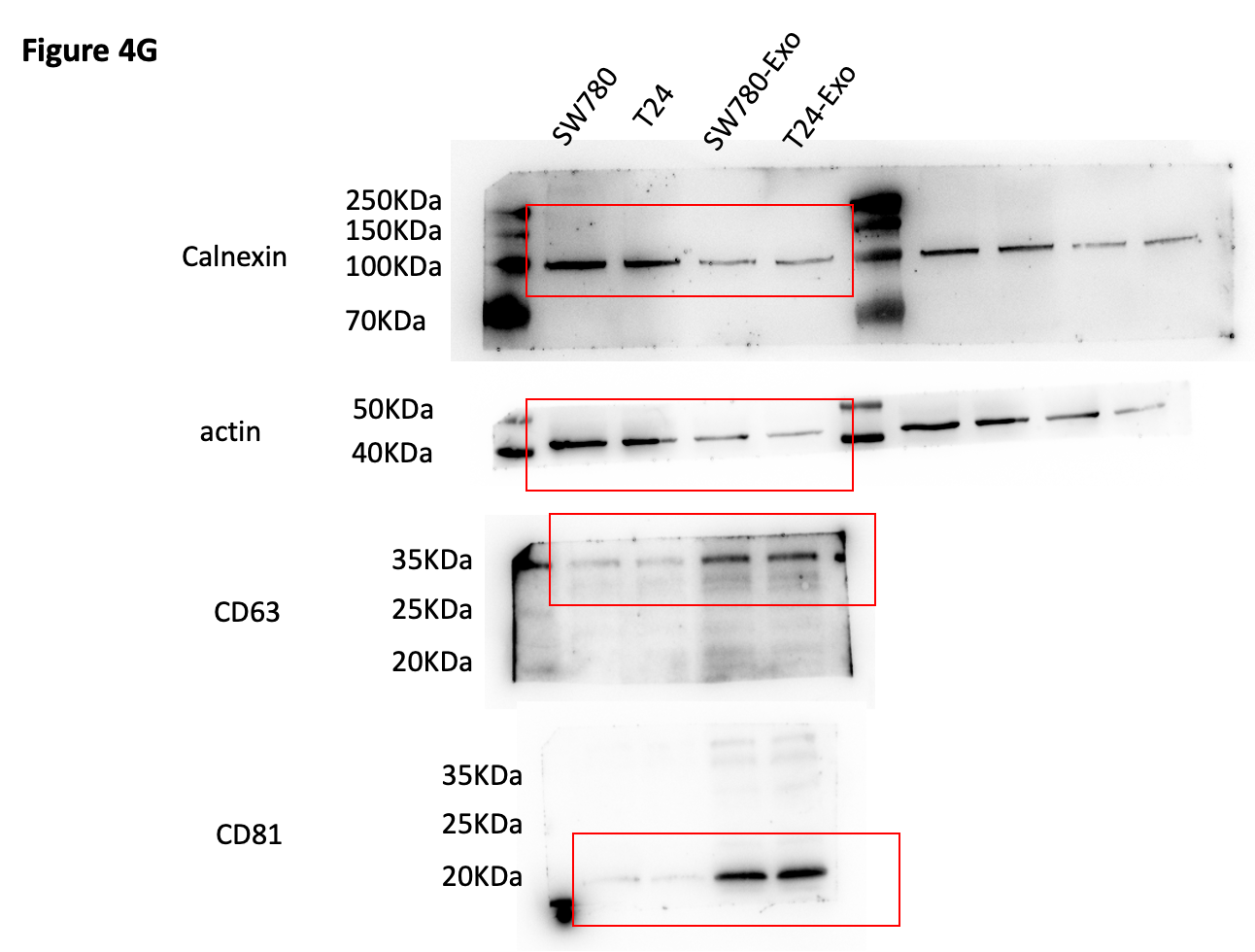
**

**
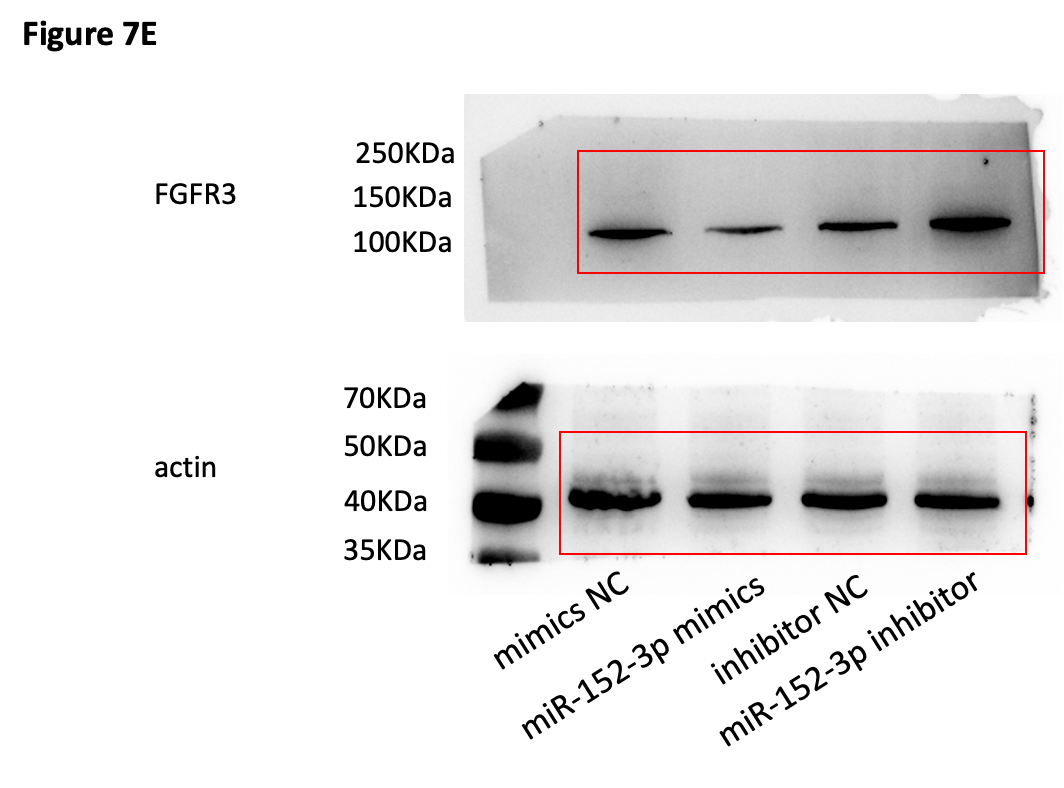
**

**
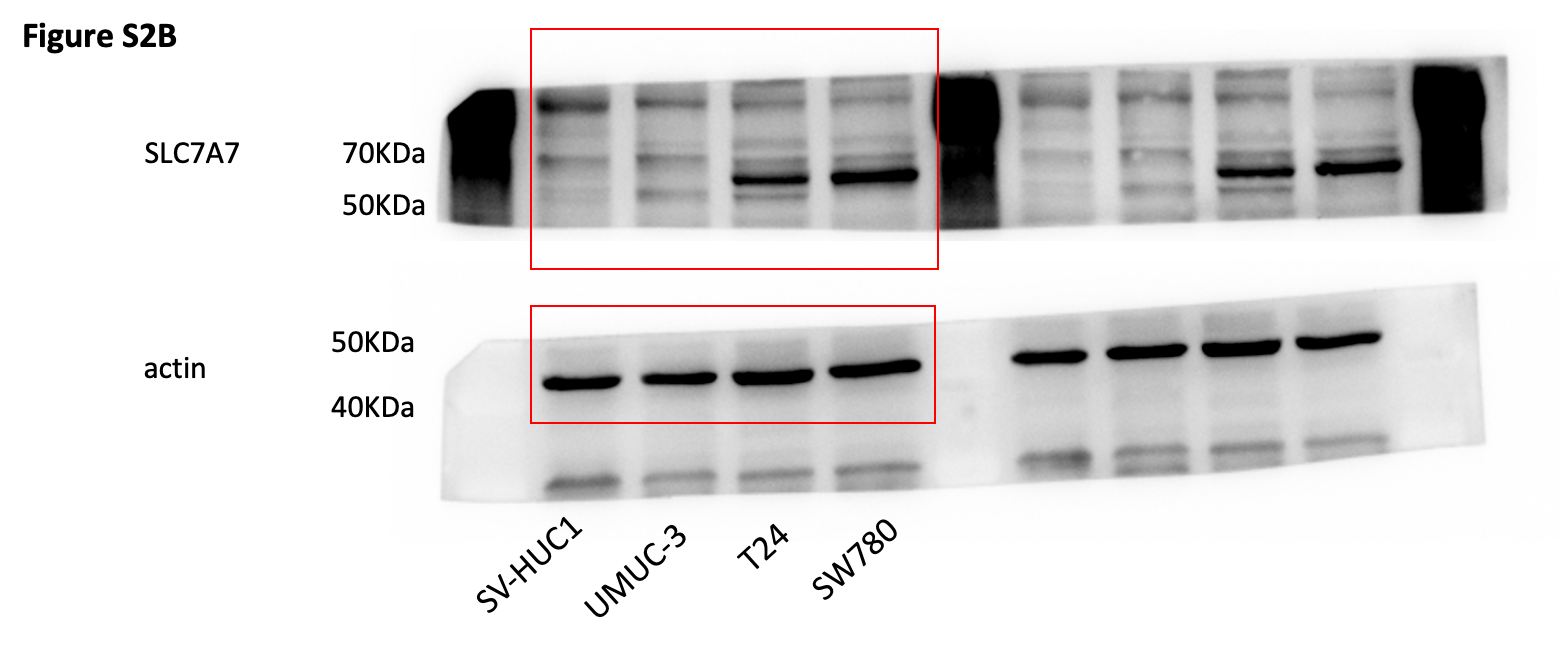
**

**
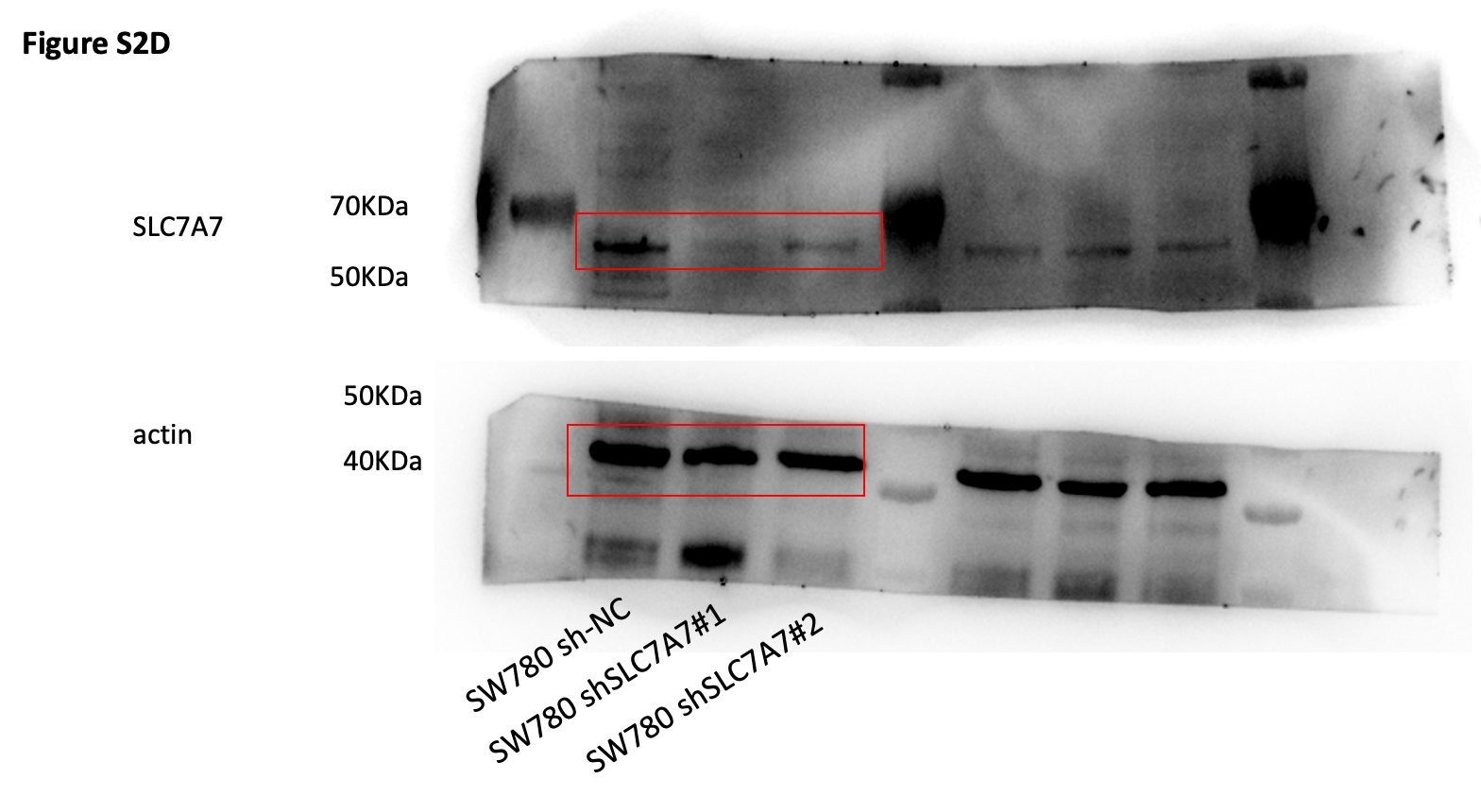
**

**
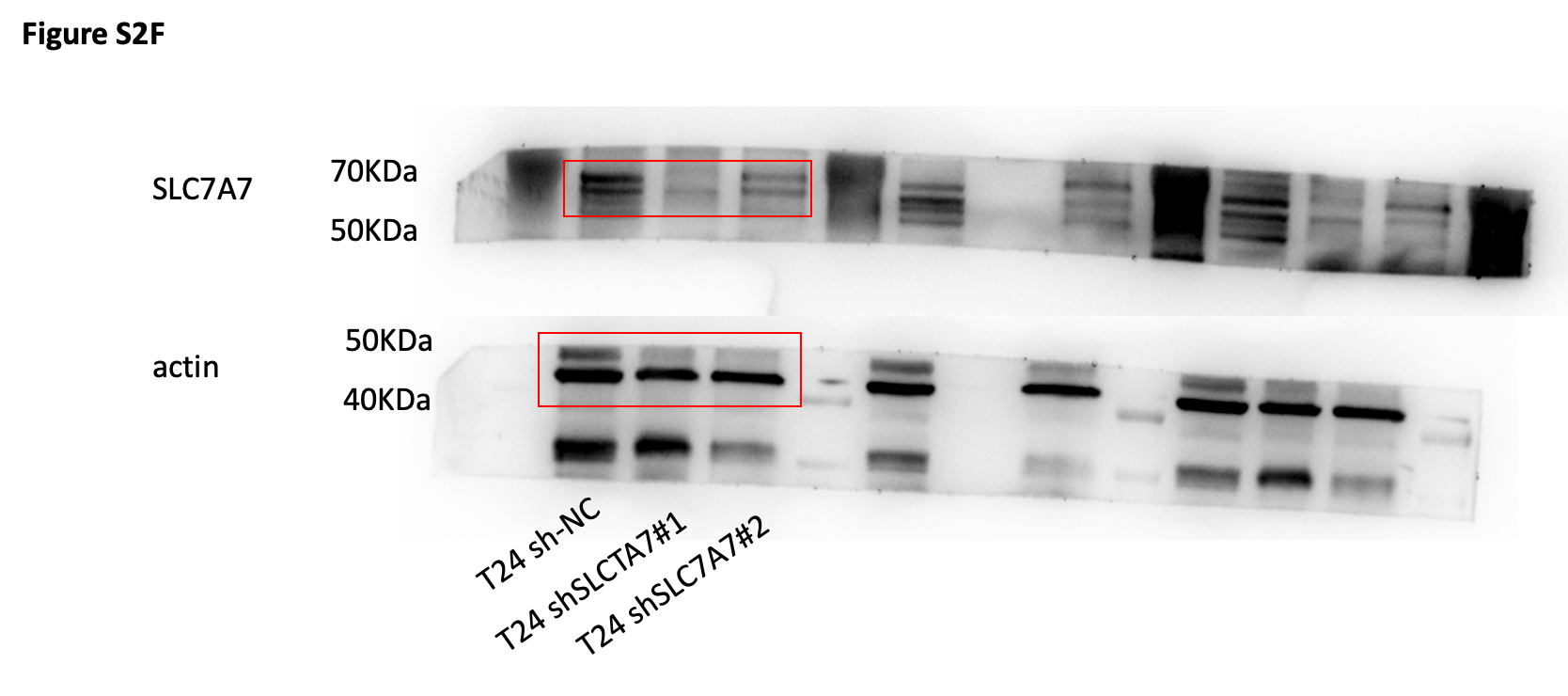
**

# Supplementary Tables

Supplementary Table 1. qPCR primers

| Amplicons | Forward Primer (5’-3’) | Reverse Primer (5’-3’) |
| --- | --- | --- |
| SLC7A7 | TGCTAAAGTATTGGCACTGATCG | AGGGCAATGTCACCCACTG |
| FGFR3 | TGCGTCGTGGAGAACAAGTTT | GCACGGTAACGTAGGGTGTG |
| β-Actin | CTCCCTGGAGAAGAGCTACGAGC | CCAGGAAGGAAGGCTGGAAGAG |
| miR-152-3p | ACACTCCAGCTGGGTCAGTGCATGACAG | CTCAACTGGTGTCGTGGAGTCGGCAATTCAGTT GAGCCAAGTT |

Supplementary Table 2. List of shRNAs

| shRNA | sequence (5’-3’) |
| --- | --- |
| Negative Control | GATCCACTACCGTTGTTATAGGTGTTCAAGAGACACCTATAACAACGGTAGTTTTTTTG |
| shSLC7A7-1 | GGTTGACAGCACTGAGTATGA |
| shSLC7A7-2 | GCATTTGTCTCTTAACCTTCA |
